# Causal effect of school-entry age on long-run family formation: Quasi-experimental evidence from 14 million individuals in Vietnam

**DOI:** 10.1093/pnasnexus/pgag119

**Published:** 2026-04-10

**Authors:** Ishaan Busireddy, Janny Liao, Dat Hoang Vu, Tam Ngo Minh Tran, Jan-Walter De Neve

**Affiliations:** School of Engineering, Stanford University, Stanford, CA 94305, USA; Harvard College, Harvard University, Cambridge, MA 02138, USA; Heidelberg Institute of Global Health, Faculty of Medicine and University Hospital, University of Heidelberg, 69120 Heidelberg, Germany; Institute of Vietnam and World Economy, Vietnam Academy of Social Sciences, Hanoi, 10000, Vietnam; New York University, Global Programs, Washington, DC 20005, USA; Accrediting Commission of Career Schools and Colleges, Accreditation Department, Arlington, VA 22201, USA; Heidelberg Institute of Global Health, Faculty of Medicine and University Hospital, University of Heidelberg, 69120 Heidelberg, Germany; Division of Global Health Management and Policy, School of Public Health, San Diego State University, San Diego, CA 92182, USA

## Abstract

School-entry age has been suggested to affect human capital development. Little is known, however, about the impacts of school-entry age on long-run outcomes, particularly in lower-income countries where most children and adolescents worldwide reside. We exploit a natural experiment based on school-entry rules in Vietnam to examine the causal effect of school-entry age on educational outcomes and family formation. Data on children's school-entry age, education, childbearing, marriage, and mortality in the next generation are extracted from the longitudinal Young Lives study conducted between 2001 and 2016 (*n* = 10,746) and the Population and Housing Censuses of 1989, 1999, and 2009 (*n* = 14,251,279). In adolescence, children who enter school at an older age stay in school longer and delay childbearing and marriage. In adulthood, women who enter school at an older age are 3.1 percentage points less likely to have given birth (*P* < 0.001, 95% CI: 3.0–3.3), have 0.1 fewer children (*P* < 0.001, 95% CI: 0.09–0.10), are 2.4 percentage points less likely to marry (*P* < 0.001, 95% CI: 2.3–2.5), and are 8% less likely (0.4 percentage points, *P* < 0.001, 95% CI: 0.3–0.5) to experience the death of a child. Decomposition analyses suggest that incapacitation mechanisms may play a larger role in shaping family formation outcomes than human capital pathways. These findings demonstrate how subtle shifts in early institutional timing can have profound and enduring impacts, with implications for education, health, and social policy globally.

Significance statement Most of the world's children live in lower-income countries, yet little is known about how early education policies shape their long-term life outcomes. Leveraging a natural experiment in Vietnam and large-scale longitudinal and census data, this study finds that entering school at an older age has lasting effects on education, family formation, and the likelihood of experiencing child mortality. These results reveal that even small shifts in school-entry policy can produce far-reaching consequences for human capital and population health, underscoring the critical role of early institutional timing in shaping life trajectories.

## Introduction

Most governments mandate age thresholds to enter public school. These policies set up a threshold rule for enrollment eligibility into grade 1 of primary school. Due to the use of age cutoff dates, the oldest children are on average 1 year older compared with the youngest children in their grade 1 cohort. Age differences at grade 1 entry exert enduring effects on personal development and environmental context. On the one hand, earlier entry into school might offer cognitive benefits and accelerate social interactions and integration (1). Earlier school entry may also increase exposure to school-based health programs (such as daily meals (2, 3) or mental health screening (4)) and offset expenditures for childcare among their parents (5–7). On the other hand, later entry into school may boost maturity and physical strength and reduce vulnerability to adverse school exposures (such as bullying) and peer pressure to engage in risky health behaviors (such as substance use or unprotected early sexual intercourse) (8). Late starters can also potentially spend more time in early childhood education programs prior to primary school. In Vietnam, a 1-year increase in early childhood education has been suggested to improve literacy and numeracy skills in adolescence by 20–30 percentage points, respectively (9). Empirical research is therefore needed to understand the full range of impacts of school-entry cutoff dates on human capital development and how these impacts affect life course trajectories in different contexts.

The impacts of age thresholds have been well documented in high-income settings (8, 10, 11). Research suggests that school-age entry policies can improve student achievement in the short term, but the long-term impacts are less well understood (12). Several recent literature reviews also point to a lack of evidence from low- and middle-income countries (12–14). An evidence map from 2022 identified one study on this policy topic from low- and middle-income countries out of over 40 studies (Text S1). For example, although in countries such as India alone over 200 million children attend school, little evidence is available from Asia. Similarly, a literature review from 2026 identified one published study from Africa, where the median age of the population is about 19 years (13, 15). In addition, the limited evidence from low- and middle-income countries tends to mirror evidence from high-income settings by focusing on selected outcomes studied in high-income countries. For example, at least three studies from Brazil and Türkiye have examined impacts on attention-deficit/hyperactivity disorder (16–18), building on findings from high-income settings (10). However, few studies from Brazil and Türkiye are available on adolescent childbearing and marriage, which remain relatively prevalent in the region, particularly among families with a lower socioeconomic status. Adolescent childbearing and marriage have been linked with adverse educational and maternal and child health outcomes (19–21). Low- and middle-income countries are inadequately represented in the existing literature, even though over 90% of children globally start primary school and grow up in low- and middle-income countries.

Here, we present one of the most extensive studies, to our knowledge, on the long-term impacts of school-entry age in lower-income countries. We first use longitudinal data from the Young Lives Study in Vietnam, which followed 3,000 children until adulthood, to document the impact of a December 31 eligibility cutoff on age at school entry. We then triangulate our findings with census data to document short- and long-term impacts of being born after the eligibility cutoff. We compare long-term outcomes of individuals who were eligible to start school later in childhood as a result of the school-entry age policy (akin to the “treatment group” in a randomized controlled trial) to the long-term outcomes of individuals who were not eligible (akin to the “control group”) (22). Because parents have limited control over the exact timing of the month of birth of their children, we can compare the children's long-run outcomes as if they were randomized into treatment and control groups by the school-entry age policy (23). Using a regression-discontinuity design, we estimate impacts around the school-entry age cutoff under the assumption that individuals born just before and after the cutoff are similar except for their eligibility to enroll in primary school during childhood. We further test this assumption underpinning causality by showing that children on either side of the cutoff are similar regarding background characteristics (such as birthweight) and placebo outcomes in adulthood (such as measured height). Lastly, to assess the robustness of our findings, we use an alternative empirical approach. Specifically, we incorporate household fixed effects into our baseline regression-discontinuity specification to control for all confounders at the level of the household or higher. We conclude that children who enter school at an older age stay in school longer and delay family formation in adolescence. In adulthood, women who enter school at an older age have fewer children, are less likely to marry or cohabitate, and are less likely to experience the death of a child.

## Methods

### Data sources and study population

#### Young Lives study

Longitudinal data on sociodemographic outcomes were extracted from the Young Lives data of Vietnam (24). This dataset contains information on about 3,000 children who have been surveyed once every 3–4 years since 2001 (25). Round 1 of the study surveyed two birth cohorts of children, including 1-year olds (born in 2001–2002) and 5-year olds (born in 1994–1995). Round 5 surveyed them when they were between 15- and 23-year olds. The younger children were tracked from infancy to their mid-teens, and the older children were tracked through adulthood, when some became parents themselves. Data were collected from families and directly from the children themselves. Strengths of the Young Lives data include the availability of data on early-life exposures and the ability to link these early-life exposures to educational and health outcomes up until early adulthood. Attrition rates in the Young Lives data are low. Over 90% of children in round 1 were followed up in round 5 (25). Information on children's exact dates of birth in the Young Lives data was classified as protected personal data and not made available to researchers. We therefore infer month of birth using the exact date of interview (variable *dint*) and age in months (variable *agemon*). Age in months was constructed by the Young Lives data team based on the child's exact date of birth and interview date. Exact dates of birth were recorded during household interviews and verified where possible using official documentation. In Supplementary analyses, we also assess robustness using alternative data sources that contain exact dates of birth. We limited the study population to children with complete data on school-entry age, yielding a final sample of 2,838 respondents, including 10,746 observations over time.

#### Vietnam population and housing census

To explore whether our findings may generalize to Vietnam more broadly and to determine longer-term impacts, we additionally extracted data from the Vietnam Population and Housing Censuses of 1989, 1999, and 2009 through the Integrated Public Use Microdata Series (IPUMS) (26). The censuses were conducted by the Bureau of the Central Steering Committee, General Statistics Office, Vietnam, using systematic stratified sampling to create random 5% (census 1989), 3% (census 1999), and 15% (census 2009) samples of the population universe. Data on month of birth and our outcomes for education and family formation were available for 99% of eligible respondents aged 5–49 years, yielding a total sample of 14,251,279 individuals. IPUMS harmonizes variables across censuses so that the same codes have the same meaning across all censuses. When using the pooled census data, we also added indicators for census year in all analyses to control for potential differences in measurement across census years and period effects. In Supplementary analyses, we also analyzed each census separately. The Vietnam Population and Housing Census of 2019, provided by IPUMS International, does not contain data on the month of birth and is therefore not included in our analyses. Additional details on these data sources and study population are available in Text S2.

### Enrollment age

In Vietnam, Education Law stipulates that children start school in September of the calendar year in which they turn 6 years of age (27). Children therefore need to be 6 years old by the cutoff of December 31 to enter grade 1 in September that school year. The school year officially starts in the first week of September and runs until the end of May the following calendar year. Primary schooling in Vietnam is compulsory, and universal primary education has been achieved (28). As a result of the school-entry age policy, children who are born just before December 31 start school 1 year earlier compared with children who are born just after December 31. Parents are required to register their children for grade 1 at local schools, and the registration process typically includes providing documents such as birth certificates and proof of residency. The policy has been suggested to be in place since at least 1945, when Vietnam became independent. We normalize the month of birth as the number of months before and after the December 31 cutoff (29). Additional information on the education system and context in Vietnam is presented in Text S3.

### Outcome measures

Our main outcomes in the Young Lives data were current school attendance, time spent in school (hours), ever having had a child, and ever having married or cohabited. Data on education were available for both the younger and older birth cohorts included in the Young Lives Study. Data on childbearing and marriage or cohabitation were only available for the older birth cohorts (ages 15–23 years). Main outcomes in the census data included school attendance, total years of schooling completed, literacy, ever having given birth among women, total number of children among women, number of own children living in the household, and ever having married or cohabited. Childbearing in the census represents the number of children ever born to each woman of whom the question was asked. In most samples, women were to report all live births by all fathers, regardless of whether the child was still living. Fieldworkers were instructed to ask the question directly to women and not indirectly via their husbands or other household members. The 1989 and 1999 samples report the number of children born directly in the questionnaire. The 2009 sample is constructed from the number of children at home, elsewhere, and those who died. In all samples, the question on childbearing was asked to all women aged 15–49 years.

### Control variables

Controlling for covariates in our education policy analysis may reduce the variance and mitigate small biases associated with including observations that are further apart from the December 31 threshold. In our analyses using Young Lives data, we controlled for month of birth, age (in years), and gender. In Supplementary analyses, we also ran models both without control variables and with additional control variables such as categorical indicators for age (rather than a continuous age variable); linear terms for year and month of birth; as well as an indicator for Young Lives birth cohort—the younger (born 2001–2002) and older cohorts (born 1994–1995). When using the census data, we controlled for age (in years) and indicators for census year (period effects). The availability of several censuses allowed us to generate variation in age for a given birth cohort. By simultaneously controlling for age and census year, we also controlled for birth cohort effects (since birth cohort equals census year “minus” a respondent's age). In sensitivity analyses, we also added additional control variables, including indicators for age (rather than a continuous age variable) and indicators for ethnicity.

### Statistical analyses

We analyzed the school eligibility cutoff in two steps. First, we used data on school-entry age from the longitudinal Young Lives Survey to determine whether cohorts who were born just after the December 31 cutoff had a higher school-entry age compared with cohorts who were born just before the cutoff (“first stage”). We estimated the impact of the eligibility cutoff on school-entry age using multivariable ordinary least squares (OLS) regression models. We used several model specifications, including (i) without control variables; (ii) with age (continuously) and a gender dummy; (iii) with age (continuously), a gender dummy, and month of birth (continuously); (iv) with age dummies, a gender dummy, and month of birth (continuously); and (v) year of birth (continuously), a gender dummy, and month of birth (continuously). Second, we employed a regression-discontinuity design (RDD) exploring the December 31 school-entry cutoff established in Vietnam since the 1940s (15, 30, 31). We assessed the intention-to-treat (ITT) effect of being born after the school-entry age eligibility cutoff on educational outcomes and family formation in multivariable regression-discontinuity linear probability models. In our base specification, we fit a centered linear trend (shown as *β*_1_ in Eq. 1) and estimate the difference at the threshold (shown as *β*_2_), the standard linear regression-discontinuity model in the literature (32). In Supplementary analyses described below, we also consider an interaction between the centered variable for month of birth and the indicator for the eligibility cutoff. The discrete nature of the assignment variable and limited support (±6 months) on either side of the cutoff limit the computation of optimal bandwidth. In Supplementary analyses, we therefore also explore generalizability to different bandwidths.


(1) $$E[Y_{i}|\mathrm{MO}B_{i}]=\beta_{0}+\beta_{1}(\mathrm{MO}B_{i})+\beta_{2}1[\mathrm{MO}B_{i}>December]$$

### Supplementary analyses

In addition to the sensitivity analyses described above, we conducted several additional analyses to generate further confidence in the robustness of our results. First, one possible threat to the validity of our results may be seasonality in births or manipulation in the month of birth around the cutoff. Causal inferences on an exposure based on month of birth could be undermined by selection based on parental characteristics, manipulation of birth dates, or early-life exposures. Sophisticated parents, for example, may time the birth of their children based on the school-entry age cutoff (33). To rule out manipulation of birth dates, we therefore show the distribution in months of birth around the cutoff. To rule out selection and confounding by early-life exposures, we also assess for balance at the threshold in parental characteristics. We plot maternal characteristics by children's month of birth, including maternal educational attainment, maternal age, and antenatal care (number of antenatal care visits and vaccinations) as well as children's birthweight (grams), using data from the Young Lives Study. Second, we conducted a placebo test. To assess whether respondents who were born before and after the cutoff were similar in predetermined characteristics, we plotted measured height by month of birth. Height is largely determined by age 5 (prior to entering school) and should, therefore, not be affected by the school-entry policy in Vietnam (23). Third, we replicated our results using alternative specifications of our sample, including when using shorter windows around the December 31 cutoff (using ±5, ±4, ±3, as well as ±2 months of birth around the December 31 cutoff as opposed to ±6 months of birth around the cutoff) and analyzing each census separately. Fourth, the Vietnam War (1955–1975) may have impacted educational processes. We therefore restricted the sample to respondents born in or after 1970 to focus on respondents who entered primary school after the war. Fifth, as an alternative indicator for childbearing, we explored impacts of school-entry age on the probability of experiencing the death of a child. Teenage mothers, for example, may face higher risks of adverse maternal and birth outcomes during pregnancy and childbirth compared with mothers who delay childbearing (19–21). We therefore assessed whether women born after the cutoff were less likely to report adverse child outcomes compared with women who were born before the cutoff using child survival data in the census (34). Sixth, we considered an alternative empirical approach. To do so, we added household fixed effects to our base specification, eliminating any potential between-household confounders. Lastly, each of the surveys described above contains information on the month of birth of all household members but does not provide data on the exact date of birth to researchers. As a robustness check, we therefore extracted data on the continuous day of birth of all children of eligible mothers as part of a woman's full birth history using data from the Vietnam Multiple Indicator Cluster Survey (35). Because eligible women in the Multiple Indicator Cluster Survey are ages 15–49 years, data on exact dates of birth were available for the next generation ages 0–34 years. To assess the loss of information from going from day of birth to month of birth, we compare results when using 3-day bins or monthly bins with quadratic fits on each side of the December 31 cutoff.

## Results

### Descriptive statistics

Table 1 lists our main data sources and sample specifications. In the Young Lives Study (round 5), 1,867 children (67.6%) were part of the Young Lives younger cohort born between 2001 and 2002, and 893 children (32.4%) were part of the Young Lives older cohort born between 1994 and 1995. Approximately 72.3% of the children resided in rural areas, and most (68%) had attended at least some pre-primary education. At the time of round 5, 1,519 children (81.4%) in the younger cohort were still enrolled in school, compared with 163 (18.3%) in the older cohort. Among the older cohort, 276 (30.9%) had been married or cohabited, and 201 (22.5%) had become parents. Among children with complete data on measured height and weight, the average body mass index in the pooled sample was 19.7 kg/m^2^ and ranged from 12.6 to 40.5 kg/m^2^. In the younger cohort, 222 children (11.9%) were stunted (defined as having height for age *z* scores <2 SDs below the median of a reference population of the same age and gender) and 170 children (9.1%) suffered from overweight or obesity (defined as having a BMI for age *z* score of one above the median of the reference group). In the pooled census data, 9,925,826 respondents aged 5–49 years (69.7%) resided in rural areas. Among respondents aged 15–23 years, 1,117,038 (33.2%) were currently attending school. Among women in this age group, 303,857 (18.3%) had ever given birth, and 446,332 (26.4%) had ever married or cohabited. For men aged 15–23 years, 201,867 (12.0%) had ever married or cohabited. Among census respondents aged 24–49 years, the average total years of schooling completed was 7.5 years for both genders, and women had an average of 2.2 children ever born.

**Table 1:** Main data sources and sample specifications.

Data source	Year	Sample	*n*
Young Lives Study cohorts, Vietnam round 5	2016–2017	Ages 14–23	2,760
Young Lives Study cohorts, Vietnam round 4	2013–2014	Ages 11–20	2,645
Young Lives Study cohorts, Vietnam round 3	2009–2010	Ages 7–16	2,730
Young Lives Study cohorts, Vietnam round 2	2006–2007	Ages 4–13	2,611
Vietnam Population and Housing Census, 15% sample	2009	Ages 5–49	10,484,262
Vietnam Population and Housing Census, 3% sample	1999	Ages 5–49	1,837,309
Vietnam Population and Housing Census, 5% sample	1989	Ages 5–49	1,929,708

The data used in this study includes all respondents in the Young Lives Study and respondents aged 5–49 in the census data, with complete information on month of birth and age.

### School-entry age by month of birth

**Figure 1:**
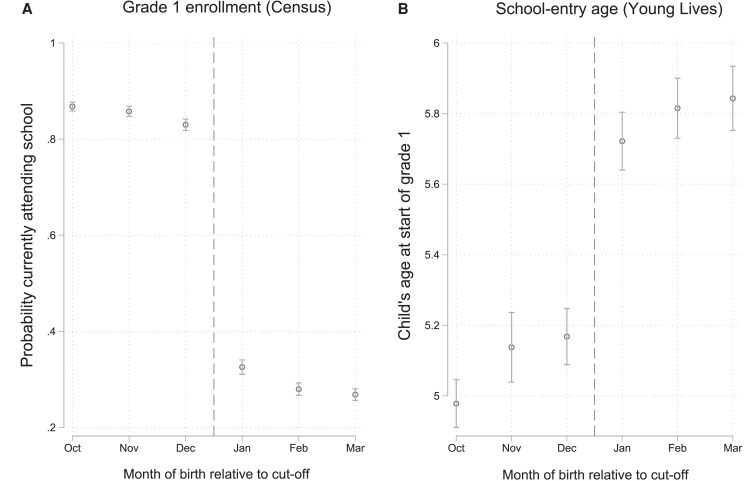
Entry into primary school in Vietnam. a) The probability of currently attending school among children born between October 1992 and March 1993 using data from the Vietnam Census of 1999 (*n* = 26,859). b) Children's age at the start of grade 1 (years) for the Young Lives Study of Vietnam cohorts ages 15–23 years using data from round 5 (*n* = 1,334). School-entry age was defined as “Child's age at start of grade 1” and was calculated by the Young Lives Study team using exact date of birth and reported schooling histories. All participants in the Young Lives sample attended at least some primary school by ages 15–23 years and thus had data on our exposure. The month of birth was defined as the month relative to the school-entry age cutoff. In Vietnam, the school-entry age cutoff is defined as December 31 (shown as a vertical dashed line). Respondents who were born just after the school-entry age cutoff were on average ∼0.7 years older at the start of grade 1 compared with respondents who were born just before the cutoff.

Figure 1 shows entry into primary school by month of birth in Vietnam. Figure 1a shows the probability of attending grade 1 by month of birth among children aged 6 years using census data. Consistent with the Education Law in Vietnam, there is a discontinuity in the probability of enrollment in grade 1 by month of birth. Children who were born just after the December 31 cutoff were on average less likely to be in school compared with children who were born just before the cutoff. In Fig. 1b, we show school-entry age by month of birth using data from the Young Lives Study for the same birth cohorts (born after 1992). Children born between January and March are on average 5.8 years old, whereas children born between October and December are on average 5.1 years old, a difference of about 0.7 years. We observe similar jumps in entry into primary school for the different Young Lives cohorts and when using different census rounds, suggesting that the policy was indeed a consistent feature of the education system in Vietnam (Text S3). We note that the difference in school-entry age shown in Fig. 1b is <1 year because some children born January–March start primary school a year early when they are 5, and some children enter primary school late despite the legal requirement. The relationship between being born after December 31 and school-entry age persisted after controlling for a wide range of control variables, including continuous measures on age, year of birth, month of birth, as well as a dummy for the Young Lives birth cohort. According to results from multivariable OLS models, being born between January and March was associated with a 0.5- to 0.8-year increase in school-entry age in the Young Lives data (Table S1).

### Impacts on school attendance and early family formation

**Figure 2:**
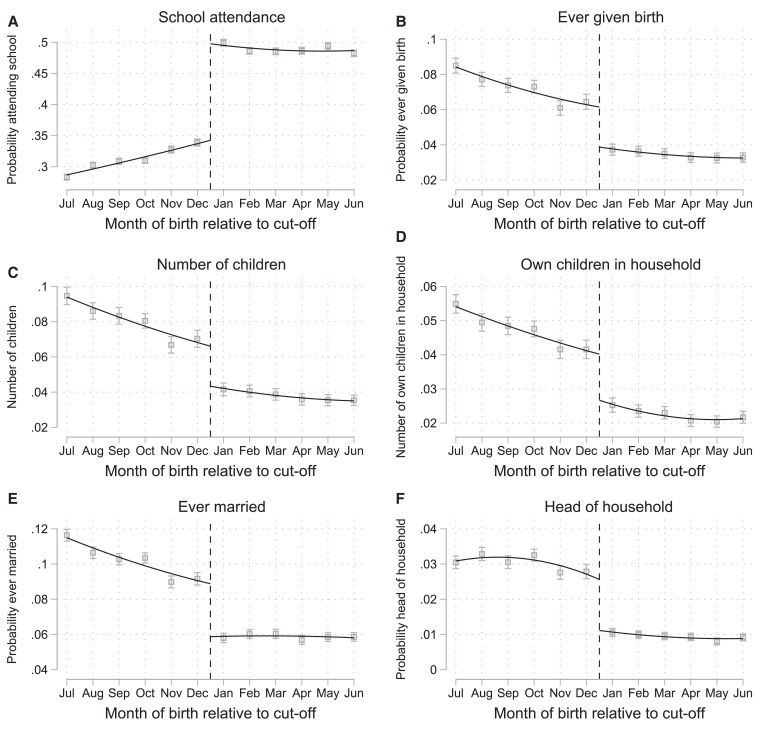
School attendance and early family formation by month of birth. The figure shows means and 95% CIs for each month of birth, superimposed with quadratic regression-discontinuity estimation using the *rdplot* command in Stata (36), among respondents turning 18 years in the Vietnam Census data. a) The probability of currently attending school (both genders). b) The probability of ever having given birth (women). c) The number of children (women). d) The number of own children in the household (both genders). e) The probability of being ever married or cohabiting (both genders). f) The probability of being head of household (both genders). Respondents were born either between July 1970 and June 1971 (census 1989), between July 1980 and June 1981 (census 1999), or between July 1990 and June 1991 (census 2009). Data from the Vietnam Population and Housing Census of 1989, 1999, and 2009 (*n* = 397,342).

In Fig. 2, we illustrate the unadjusted relationship between our main outcomes and month of birth during the period of late adolescence. Although children born between July and December entered school earlier, children born between January and June (who were eligible to enter school late) were more likely to still be in school. Adolescents born after the eligibility cutoff were considerably more likely to still be attending school by age 18 years, were less likely to have given birth (women), had fewer children, were less likely to be married, and were less likely to be the head of household. Being born after the cutoff reduced the probability of being head of household by age 18 years by more than half. Table 2 shows ITT regression results using data from the Young Lives Study, controlling for age and gender. Children born after the cutoff were 3.3 percentage points more likely (*P* = 0.013, 95% CI: 1.0–5.8) to be currently in school; spent 0.21 h per day more (*P* = 0.002, 95% CI: 0.08–0.33) studying at home; were 7.5 percentage points less likely (*P* = 0.031, 95% CI: 1.0–14.3) to have had a child; and were 9.9 percentage points less likely (*P* = 0.007, 95% CI: 2.6–17.1) to have married or cohabitated. In Table 3, we show estimates using pooled census data for respondents aged 15–23 years. Consistent with results from Young Lives data, being born after the eligibility cutoff was associated with a 6.6 percentage point increase (*P* < 0.001, 95% CI: 6.4–6.8) in school attendance and a 4.6 percentage point decrease in the probability of marriage or cohabitation (*P* < 0.001, 95% CI: 4.4–4.7). Women also saw a 5.1 percentage points reduction (*P* < 0.001, 95% CI: 4.9–5.3) in childbearing. These effects were substantive. Given a baseline prevalence of childbearing of 18.8% among young women born before the eligibility cutoff, these results suggest that being born after the cutoff decreased the probability of childbearing by more than a quarter among women born after the cutoff.

**Table 2:** Effect of being born after the school-entry age cutoff on school attendance and early family formation, Young Lives data (RDD).

Intention-to-treat model	(1)	(2)	(3)	(4)	(5)
Dependent variable (DV)	Currently attending school (1 = yes)	Hours per day spent at school	Hours per day spent on studying outside of school	Has son or daughter (1 = yes)	Ever married or cohabited (1 = yes)
Predictor					
Born January–June (1 = yes)	0.033**	0.338***	0.206***	−0.075**	−0.099***
	(0.013)	(0.088)	(0.065)	(0.035)	(0.037)
Additional covariates					
Month of birth	✓	✓	✓	✓	✓
Age (years)	✓	✓	✓	✓	✓
Gender	✓	✓	✓	✓	✓
Sample					
Young Lives Study, round 5	✓	✓	✓	✓	✓
Young Lives Study, round 4	✓	✓	✓	✓	✓
Young Lives Study, round 3	✓	✓	✓	—	—
Young Lives Study, round 2	✓	✓	✓	—	—
Mean DV, July–December birth cohorts	0.811	4.5	2.0	0.192	0.221
Observations	10,746	10,746	10,739	1,690	1,690
Respondents	2,838	2,838	2,837	897	897

Data source: Young Lives data, 2006–2017. Columns 4–5 include respondents in the Young Lives Study with complete data on childbearing and marriage (ages 18 to 23 years). The month of birth variable is continuous and centered as the month of birth—6.5; age (in years) is a continuous variable, and gender is a dummy variable. Data on fertility and marriage were unavailable for the younger birth cohorts in the Young Lives Study (rounds 2 and 3). Robust unclustered SEs in parentheses (***P < 0.01, **P < 0.05, *P < 0.1).

**Table 3:** Effect of being born after the school-entry age cutoff on school attendance and early family formation, census data (RDD).

Intention-to-treat model	(1)	(2)	(3)	(4)	(5)
Dependent variable (DV)	Currently attending school (1 = yes)	Ever given birth (1 = yes)	Number of children ever born	Number of own children in household	Ever married or cohabited (1 = yes)
Predictor					
Born January–June (1 = yes)	0.066***	−0.051***	−0.070***	−0.047***	−0.046***
	(0.001)	(0.001)	(0.002)	(0.001)	(0.001)
Additional covariates					
Month of birth	✓	✓	✓	✓	✓
Age (years)	✓	✓	✓	✓	✓
Census year	✓	✓	✓	✓	✓
Gender	✓	—	—	✓	✓
Sample					
Ages 15–23	✓	✓	✓	✓	✓
Female	✓	✓	✓	✓	✓
Male	✓	—	—	✓	✓
Mean DV, July–December birth cohorts	0.323	0.188	0.243	0.157	0.196
Observations	3,367,641	1,656,126	1,656,126	3,367,641	3,367,641
R-squared	0.234	0.202	0.182	0.143	0.212

Data source: Sample of respondents aged 15–23 at the time of the survey in the Vietnam Population and Housing Census 1989, 1999, and 2009 with complete information on childbearing outcomes (*N* = 3,367,641). The month of birth variable is continuous and centered as the month of birth—6.5; age (in years) is a continuous variable, and census year and gender are dummy variables. Robust unclustered SEs in parentheses (***P < 0.01, **P < 0.05, *P < 0.1).

### Impacts on educational attainment and long-term family formation

**Figure 3:**
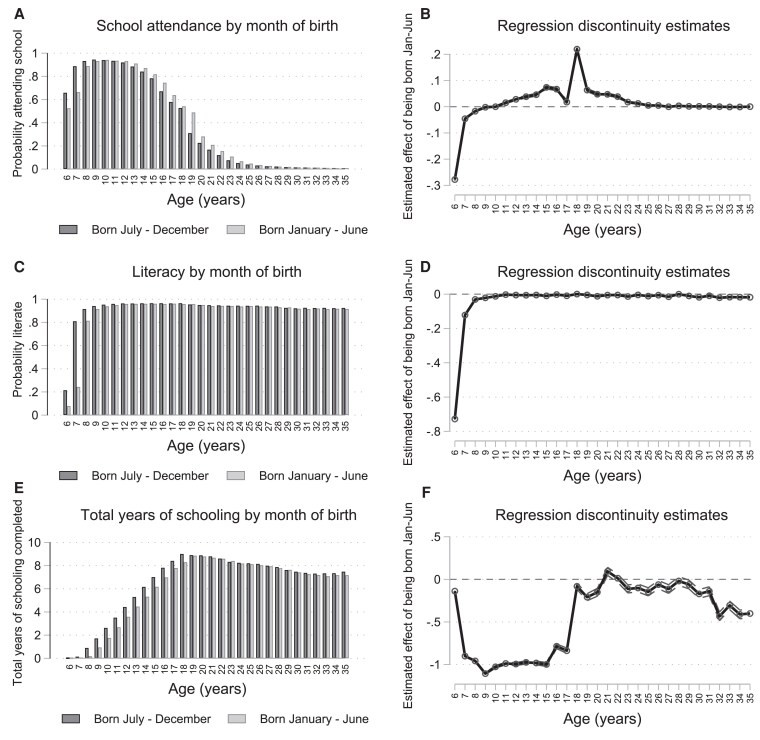
Regression discontinuity impacts on education by age. In (a), (c), and (e), we show descriptive means for our outcomes separately for respondents who are born either between July and December or between January and June using pooled census data, separately for each single-year age group. In (b), (d), and (f), we show ITT regression estimates for the effect of being born after the December 31 school-entry age cutoff on schooling outcomes, estimated separately at each age. All regression models included the treatment variable (an indicator for being born between January and June), centered month of birth, and indicators for census year. The outcomes were a binary indicator for current school attendance (a and b), literacy (c and d), and total years of schooling completed (e and f). Literacy in the census data indicates whether the respondent could read and write in any language. A person was typically considered literate if he or she could both read and write. In addition, in 1999, people with 5 or more years of schooling were considered literate. In 2009, people who completed more than primary education were considered literate. Dashed lines represent 95% CIs. The sample includes all respondents aged 6–35 years in the Vietnam Population and Housing Census of 1989, 1999, and 2009 (*n* = 10,485,714).

Figure 3a, c, and e shows descriptive means for our outcomes separately for respondents who were born either between July and December or between January and June using pooled census data, separately for each single-year age group. Figure 3b, d, and f shows coefficients from regression-discontinuity models for the effect of school-entry age (being born January–June) controlling for centered month of birth and indicators for the census year. As a first step, we show differences in educational trajectories over time to explore whether differences in school-entry age induced changes in school attendance, cognitive skills (such as measured literacy), and total educational attainment, which may provide insights into subsequent decisions around starting a family and household size. Figure 3a and b shows results for current school attendance. Consistent with the school-entry age policy, children born after the eligibility cutoff are initially less likely to be in school. However, this difference in school attendance reverses in adolescence, when children born after the eligibility cutoff become more likely to still be in school, particularly around age 18 years. The increase in school attendance in adolescence, however, does not necessarily translate into differences in human capital acquisition. Overall, we detect few differences in literacy between children born before the cutoff and after the cutoff in recent cohorts ages 18–30 years. Similarly, in terms of educational attainment, shown in Fig. 3e and f, children born after the cutoff have initially fewer total years of schooling during childhood because of the eligibility cutoff (31) but catch up with children born before the cutoff as soon as late-starters turn 18 years. Among older cohorts (around ages 30+ years), children born after the cutoff complete fewer total years of schooling (37). Figure S1 shows coefficients from regression-discontinuity models disaggregated by gender. While the impacts of school-entry age on educational outcomes are largely similar for young women and men, the implications for long-term family formation may differ by gender, which we explore further in the next paragraph.

**Figure 4:**
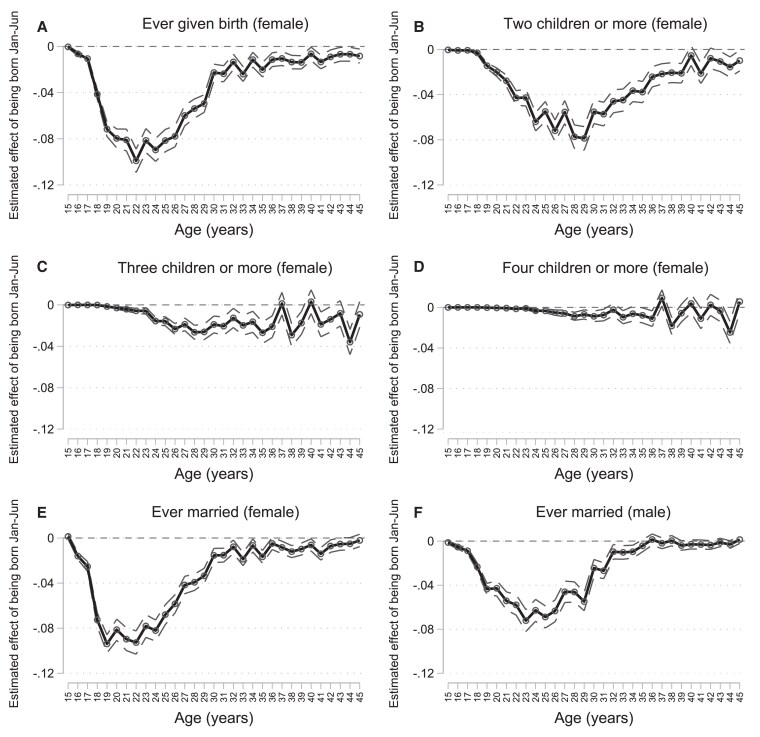
Regression discontinuity impacts on family formation by age. Figure shows ITT regression estimates for the effect of being born after the December 31 school-entry age cutoff on long-run family formation, estimated separately at each age. All regression models included the treatment variable (an indicator for being born between January and June), centered month of birth, and indicators for the census year. The outcomes were binary indicators for ever have given birth (a), have given birth to two children or more (b), have given birth to three children or more (c), have given birth to four children or more (d), and ever having married or cohabited (e and f). Childbearing in the census represents the number of children ever born to each woman for whom the question was asked. In most samples, women were to report all live births by all fathers, regardless of whether the child was still living. Dashed lines represent 95% CIs. The sample includes all respondents aged 15–45 years in the Vietnam Population and Housing Census of 1989, 1999, and 2009. (*n* = 10,485,714).

In Fig. 4, we show results for the impacts of being born after the eligibility cutoff on long-term family formation. We show differences in family formation for respondents born between July and December vs. those born between January and June using pooled census data, estimated separately for each single-year age group and gender. Similar to before, we estimate differences at the cutoff controlling for the centered month of birth and indicators for the census year. Figure 4a shows results for the probability of ever given birth. The difference in childbearing for women born between July and December vs. January and June appears by late adolescence and peaks in early adulthood by around age 22 years. Late starters are less likely to have children during adolescence, putting them at lower risk of adverse maternal and child health outcomes (19–21, 38). In Vietnam, the government has also aimed to curb population growth by imposing penalties for exceeding child limits (such as the two-child policy). We therefore show differences in the “distribution” of childbearing, including the probability of having at least two children, at least three children, or at least four children in Fig. 4b–d. Figure 4e–f shows results for marriage or cohabitation. In Table 4, we show the full output from regression-discontinuity models. In adulthood, women born after the school-entry age cutoff are 3.1 percentage points less likely to give birth (*P* < 0.001, 95% CI: 3.0–3.3) and have 0.1 fewer children (*P* < 0.001, 95% CI: 0.09–0.10). We observed the largest absolute differences for having at least two children: women born after the cutoff are 4.2 percentage points less likely to have two children or more (*P* < 0.001, 95% CI: 4.0–4.4). In the pooled sample (both genders), individuals born after the cutoff are 2.2 percentage points less likely (*P* < 0.001, 95% CI: 2.1–2.3) to marry or cohabitate. When disaggregating our results by gender, women born after the cutoff are 2.4 percentage points less likely (*P* < 0.001, 95% CI: 2.3–2.5) and men born after the cutoff 2.2 percentage points (*P* < 0.001, 95% CI: 2.1–2.4) less likely to marry.

**Table 4:** Effect of being born after the school-entry age cutoff on long-term family formation, census data (RDD).

Intention-to-treat model	(1)	(2)	(3)	(4)	(5)
Dependent variable (DV)	Ever given birth (1 = yes)	Two children or more (1 = yes)	Three children or more (1 = yes)	Number of children ever born	Ever married or cohabited (1 = yes)
Predictor					
Born January–June (1 = yes)	−0.031***	−0.042***	−0.017***	−0.095***	−0.022***
	(0.001)	(0.001)	(0.001)	(0.003)	(0.001)
Additional covariates					
Month of birth	✓	✓	✓	✓	✓
Age (years)	✓	✓	✓	✓	✓
Census year	✓	✓	✓	✓	✓
Gender	—	—	—	—	✓
Sample					
Ages 24–49	✓	✓	✓	✓	✓
Female	✓	✓	✓	✓	✓
Male	—	—	—	—	✓
Mean DV, July–December birth cohorts	0.875	0.686	0.324	2.2	0.884
Observations	3,650,035	3,650,035	3,650,035	3,650,035	7,175,064
*R-squared*	0.053	0.145	0.181	0.230	0.084

Data source: Sample of respondents aged 24–49 at the time of the survey in the Vietnam Population and Housing Census 1989, 1999, and 2009 with complete information on childbearing (*n* = 7,175,064). The month of birth variable is continuous and centered as the month of birth—6.5; age (in years) is a continuous variable, and census year and gender are dummy variables. Robust unclustered SEs in parentheses (***P < 0.01, **P < 0.05, *P < 0.1).

### Results from supplementary analyses

**Figure 5:**
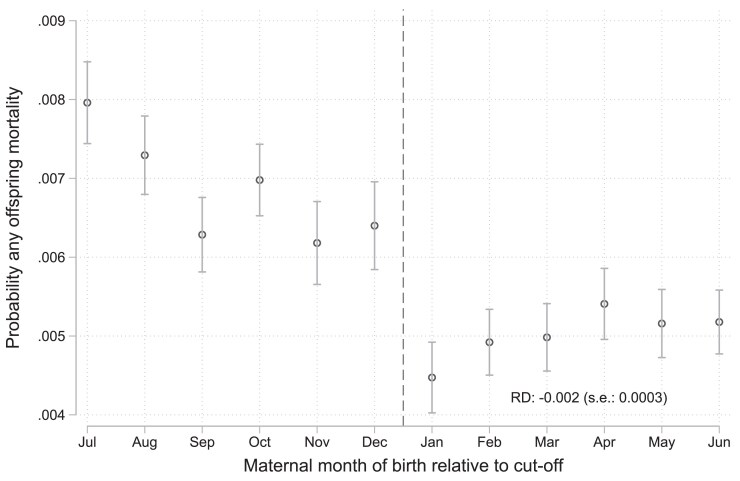
Impact on lifetime prevalence of offspring mortality (women, ages 18–23 years). The figure shows maternal lifetime prevalence of any offspring mortality by maternal month of birth. We show means and CIs for each month of birth. We also show the point regression estimate and standard error (s.e.) from a regression-discontinuity model when controlling for a continuous term in maternal month of birth, maternal age (continuously in years), and census year. In the subsample of women ages 18–23 years, mothers born after the cutoff had a 0.2 percentage point lower lifetime prevalence of experiencing any child mortality. In the subsample of women ages 24–49 years, mothers born after the cutoff had a 0.4 percentage point lower lifetime prevalence of experiencing child mortality. Additional information on specifications used in regression-discontinuity models and full regression output is shown in Table S12 in the Appendix. The outcome was defined as the proportion of mothers who have ever experienced an infant, an under 5-y-old child, or any-age child die. The sample in the figure includes all women aged 18–23 years in the Vietnam Population and Housing Census 1989, 1999, and 2009.

Figure S2 shows the distribution of month of birth around the cutoff using data from the Young Lives Study, separately by survey round and cohort. We observe no evidence of bunching at the December 31 threshold. We also find no differences in maternal educational attainment (years), maternal age at birth (years), number of antenatal visits by mothers, number of antenatal vaccinations among mothers, or children's gender by children's month of birth (Fig. S3 and Table S2). We additionally find no differences in birthweight for children born on either side of the threshold. These findings support the validity of our identification strategy. Children born in July–December vs. January–June had similar parents, grew up in similar households, and had similar early childhood anthropometric measurements. They look similar on all assessed measures until they start primary school. Figure S4 shows results from a placebo test using data on measured height. Height is similar for respondents born before and after the cutoff. In our main analysis, we limited the sample to children born 6 months before and after the cutoff. In Table S3, we show ITT results when using alternative sample specifications, including when using windows of ±5, ±4, ±3, and ±2 months of birth around the cutoff. We also present results when using additional control variables (Table S4), limiting the sample to respondents who entered school after the Vietnam War (Table S5), analyzing each census separately (Tables S6–S8), and including an interaction between the centered month of birth variable and the eligibility indicator (Tables S9–S11). Our results are consistent across all those alternative specifications. Figure 5 shows the binned scatter plot for lifetime prevalence of offspring mortality by maternal month of birth. We find that, possibly due to both lower childbearing reported above and lower child mortality, mothers born after the cutoff had a lower lifetime prevalence of experiencing mortality among their offspring. In Table S12, we show regression-discontinuity estimates for the impacts of maternal school-entry age on the lifetime prevalence of experiencing mortality, controlling for maternal month of birth, maternal age, and census year. In the subsample of women ages 18–23 years, mothers born after the cutoff had a 0.2 percentage point (*P* < 0.001, 95% CI: 0.2–0.3) lower lifetime prevalence of experiencing any child mortality, relative to a baseline prevalence of 0.8%. In the subsample of women ages 24–49 years, mothers born after the cutoff had a 0.4 percentage point (*P* < 0.001, 95% CI: 0.3–0.5) lower lifetime prevalence of experiencing child mortality, relative to a baseline prevalence of 5.1%. In Table S13, we add household fixed effects to our base regression-discontinuity specification, which allows us to assume that the differences we observe between early- and late-starters are likely not due to unobserved confounders at the household level such as parental efforts to optimally time their birth. Although these results are not directly comparable to our main results since they are de facto only estimated using the sample of households with more than one eligible respondent, our results are consistent. In within-household analyses, women born after the school-entry age cutoff are 4.6 percentage points less likely to give birth (*P* < 0.001, 95% CI: 3.8–5.4) and have 0.2 fewer children (*P* < 0.001, 95% CI: 0.17–0.21). Lastly, in Fig. S5, we show our first stage results using either the exact day of birth or month of birth in the Vietnam Multiple Indicator Cluster Surveys data. The loss of information from going from day of birth to month of birth seems limited. These results are also qualitatively similar to those presented in Fig. 1.

## Discussion

The age at which children enter primary school has been suggested to affect human capital development later in life, but results for the long-term impacts of school-entry age are mixed and little evidence is available from low- and middle-income countries, where most children and adolescents go to school and start a family (12–14). Prior studies have also frequently relied on data from cross-sectional surveys to assess impacts of school-entry age (14). Here, we sought to determine the impacts of school-entry age by triangulating evidence from multiple datasets, including longitudinal data on children in the Young Lives Study who were followed until adulthood (25). In adolescence, children born after the school-entry eligibility cutoff stay in school longer and delay childbearing and marriage. In adulthood, women born after the school-entry age cutoff are less likely to give birth, have fewer children across the “distribution” of childbearing (i.e. including having at least one, two, three, or more children), are less likely to marry, and are less likely to experience the death of a child. The magnitude of the effect sizes identified in our study was substantive. The Young Lives Study also provided data on children's birthweight and other background characteristics, enabling us to strengthen the key assumptions underlying causal inference (Fig. S3). Our findings remained consistent across a range of supplementary analyses, including the use of a narrower birth-month window around the school-entry cutoff (Table S3), a placebo test (Fig. S4), and an alternative empirical approach (Table S13).

Differences resulting from early-life exposures, such as school-entry age eligibility, may compound over the life course, influencing not only when people have children or get married but also the quality of their relationships and long-term composition of family structures. Early family formation is relatively common in Vietnam (Fig. 2) and has been linked with reduced women's bargaining power (39), human capital development (40, 41), and adverse maternal and child health outcomes (19, 21, 38, 42). Adolescent mothers (aged 10–19 years) are at greater risk of eclampsia, puerperal endometritis, and systemic infections compared with women aged 20–24 years, and babies of adolescent mothers face higher risks of low birth weight and severe neonatal conditions (38, 43). Delaying childbearing may also have implications for choices around continuing education, employment, income, and could impact individuals’ social networks. For example, adult children provide considerable time and financial contributions to household members, and delaying or reducing childbearing may lead to fewer resources among older household members (44). This may be particularly the case in contexts where public welfare transfers are limited and older adults rely on intergenerational transfers (45). Children who start school at an older age have fewer children in adulthood, suggesting that they both delay and reduce childbearing permanently (Fig. 4).

At least three potentially competing pathways through which school-entry age may affect long-term family formation have been documented. First, as noted above, school-entry age may have relative age effects (46). Children who are relatively older than their classmates may develop more self-esteem and be less likely to be adversely influenced by their peers in school or in the workplace (47, 48). Second, school-entry age may affect human capital accumulation through its impact on cognitive skills, such as measured literacy and numeracy skills, grade repetition or school dropout, and total years of schooling completed. Third, if school-entry age affects school attendance, then school-entry age may also have “incapacitation” effects (49). Children who start school later are pushed into school longer by law—i.e. they are essentially locked up in school longer (50). Children who start school later complete their basic schooling at a later age compared with children who start school earlier. Indeed, we observe large differences in school attendance among adolescents ages 18 years (Fig. 3), which coincides with the end of high school in Vietnam (12th grade). In addition, the effect of “incapacitation” may also stem from potential sociocultural norms that consider childbearing and marriage incompatible with schooling.

To investigate how school-entry age impacts family formation, we conducted a series of regression models with an increasing set of controls to target specific mechanisms of interest. We implemented our main ITT models while additionally controlling for either current school attendance or total years of schooling completed. This allowed us to determine whether accounting for these potential pathways would reduce the size of the coefficient. We find that controlling for school attendance reduces the magnitude of the coefficient on total childbearing by ∼70% from 0.020 to 0.006 (Table S14), whereas controlling for years of schooling made little difference on the coefficient for childbearing (Table S15). These results suggest that school attendance may explain at least some of the observed effects for family formation in this age group (incapacitation effects). These results are also in line with the findings presented by Geruso and Royer (51) and Adamecz-Völgyi and Scharle (49) who attribute the effect of school-entry age on childbearing to school attendance rather than human capital effects (49, 51). Berthelon and Kruger (52) show that an increase in the length of the school day reduces risky behavior among adolescents and significantly lowers adolescent childbearing (e.g. by keeping children in school and preventing risky behaviors). In contrast, other studies find combined impacts of school attendance and years of schooling, suggesting that human capital pathways may explain at least partly the impact on early family formation in some settings (53–56).

Examining the long-term effects of school-entry age presents several methodological challenges. First, the longitudinal Young Lives data are purposely sampled and thus not nationally representative. To mitigate potential selection bias, we complemented this analysis with other national surveys from Vietnam. Results across datasets consistently estimated the effects of school-entry age on various outcomes. Second, the data do not allow for disentangling the impacts of relative vs. absolute ages. To address this, we disaggregated our results by single-year age groups, focusing on comparisons at the cutoff among respondents who differ in absolute age by only one month, thus likely being similar in maturity and biological age. Thirdly, our results are specific to the population whose enrollment decisions were affected by the policy in Vietnam. Household and school responses to school-entry policies may vary in other settings, limiting generalizability. Finally, the census data lack information on the gender and birth order of all children born to a mother. In Vietnam, strong son preference driven by social and economic factors may lead parents to continue childbearing until a male heir is born (57). Future research should examine whether school-entry age policies influence gender composition in households. For instance, if being born after the cutoff is associated with having fewer sons or daughters. Similarly, future studies could take advantage of this natural experiment to further explore the effects of relative vs. absolute age, psychological pathways, and long-term socioeconomic and health outcomes, including labor force participation, professional occupation, income, biomarkers, and medical record data.

## Supplementary Material

pgag119_Supplementary_Data

## Data Availability

This study uses two main data sources. The Young Lives Study (2001–2016) is a publicly available longitudinal dataset on child development in Vietnam. The data are accessible from the UK Data Service (http://doi.org/10.5255/UKDA-Series-2000060) upon registration and completion of a data access agreement (24). The Vietnam Population and Housing Censuses of 1989, 1999, and 2009 are available through the IPUMS International database (https://doi.org/10.18128/D020.V7.6) subject to a data use agreement (26). The census data were originally collected by the Bureau of the Central Steering Committee, General Statistics Office, Vietnam. All analyses were conducted using Stata MP v.19. All coding used in this study is available on the Harvard Dataverse repository (https://doi.org/10.7910/DVN/RJWRF9).

## Preprints

This manuscript was posted as a preprint at https://www.medrxiv.org/content/10.1101/2024.09.18.24313904v1.
