# Supplementary material for: Causal effect of school-entry age on long-run family formation: Quasi-experimental evidence from 14 million individuals in Vietnam
Source: PNAS Nexus. 2026 Apr 10;5(5):pgag119. doi: 10.1093/pnasnexus/pgag119 (PMC13147269; doi:10.1093/pnasnexus/pgag119)
Supplement: pgag119_Supplementary_Data [file pgag119_supplementary_data.pdf]

Supplementary Material

Supplement to: Causal effect of school-entry age on long-run family formation: quasi-experimental evidence from 14 million individuals in Vietnam (Busireddy et al., 2026)

Text S1. Literature on school-entry age 2
Text S2. Additional information on data sources 3
Text S3. Education context in Vietnam 5
Figure S1. Heterogeneity: impacts on education by gender 7
Figure S2. Distribution in month of birth 8
Figure S3. Balance in background characteristics 9
Figure S4. Placebo test: measured height by month of birth 10
Figure S5. School enrollment by exact date of birth 11
Table S1. First stage regression results: impacts on school-entry age (OLS) 12
Table S2. Balance: estimates for background characteristics 13
Table S3. Robustness checks: using narrower windows of month of birth 14
Table S4. Robustness checks: using additional control variables 15
Table S5. Robustness checks: respondents born in or after 1970 16
Table S6. Robustness checks: analyzing 2009 Census separately 17
Table S7. Robustness checks: analyzing 1999 Census separately 18
Table S8. Robustness checks: analyzing 1989 Census separately 19
Table S9. Robustness checks: adding (Born Jan-Jun) x Birth month (Young Lives) 20
Table S10. Robustness checks: adding (Born Jan-Jun) x Birth month (Census, ages 15-23) 21
Table S11. Robustness checks: adding (Born Jan-Jun) x Birth month (Census, ages 24-49) 22
Table S12. Impact on lifetime prevalence of offspring mortality 23
Table S13. Alternative empirical approach: within-household analyses 24
Table S14. Pathways: controlling for school attendance 25
Table S15. Pathways: controlling for years of schooling 26
References for Appendix 27

Text S1. Literature on school-entry age

We assess effects in a lower middle-income country context where factors linked to this context may shape divergent trajectories in educational processes and long-term family formation. A recent evidence map from Dhuey and Koebel (2022) graphically shows where the evidence on the impacts of school-entry age comes from. The map is available at the following link: https://wol.iza.org/articles/age-at-school-entry-how-old-is-old-enough/map. The colored countries have empirical evidence for this topic. The number on the flag indicates how many relevant academic studies address this policy question. Lower middle-income countries, however, such as Vietnam, are not represented in the existing literature. The review identified one study from an upper middle-income country. Findings from higher-income settings, however, may not generalize to low- and middle-income countries where the context is very different. Similarly, two other recent reviews find little evidence on long-term outcomes from low- and middle-income countries (Liao et al, 2023, Cavallo et al 2026).

The impacts of these policies may differ considerably in the context of poverty. Larger class sizes, student heterogeneity, and less formal training may constrain teachers' ability to teach across the full distribution of skill levels. Fewer seats in secondary and tertiary education could create bottlenecks and may reduce incentives for teachers, students, and households to invest in the lowest-performing students. Laws requiring students to stay in school up to specific ages and legal regulations on child labor may not be fully enforced. Additionally, the opportunity costs of schooling may be greater where children can work in agriculture, perform home production activities, or enter the labor force. Preventing households from making optimal decisions around schooling may carry a different penalty in lower-income countries with potentially large downstream effects. The lack of evidence is important because the existing evidence on long-term outcomes is mixed so far and, theoretically, the long-term effects may be much larger in lower-income countries, such as Vietnam, compared to higher-income countries.

Text S2. Additional information on data sources

Young Lives Study

Data on socio-demographic characteristics, schooling, and health outcomes were extracted from Young Lives data. These datasets contain information on a longitudinal study of poverty and inequality following the lives of 3,000 children in Vietnam over a 15-year period, surveyed once every 3-4 years since 2001 (Favara M et al, 2021). Round 1 of the study surveyed two groups of children, 1-year-olds (born in 2001-02) and 5-year-olds (born in 1994-1995). Round 5 surveyed them when they were between 15 and 23 years old. The younger children were tracked from infancy to their mid-teens, and the older children into adulthood, when some became parents. Data was collected from families and directly from the children themselves. The Young Lives study is not intended to be a nationally representative survey. Still, it aims to show the impact of early-life circumstances on children's later educational and health outcomes. Multistage purposive sampling was used for sample selection, with the first stage involving a selection of 20 sentinel sites. The 20 sites are located in five provinces (Ben Tre, Da Nang, Hung Yen, Lao Cai, and Phu Yen). Households in selected sites were then chosen at random.

Attrition rates in the Young Lives data are low, and over 90% of children were included in all rounds (rounds 1 to 5). We therefore infer month of birth using the exact date of interview (variable *dint*) and age in months (variable *agemon*). Age in months was constructed by the Young Lives data team based on the child's exact date of birth and interview date. Exact dates of birth were recorded during household interviews and verified where possible using official documentation. Key advantages of the Young Lives data include that it provides a variable for school-entry age (age at beginning of Grade 1) and the ability to link school-entry age to measured educational and health outcomes up until early adulthood. Round 6 was a phone survey during the COVID-19 pandemic and was not included in our analysis.

Vietnam Population and Housing Census

Data were extracted from the 1989, 1999, and 2009 Vietnam Population and Housing Censuses through the Integrated Public Use Microdata Series (IPUMS). The Censuses were conducted by the Bureau of the Central Steering Committee, General Statistics Office, Vietnam, using a systematic stratified sampling to create random 5% (Census 1989), 3% (Census 1999), and 15% (Census 2009) samples of the population universe. Data on month of birth and our outcomes for education and family formation were available for 99% of eligible respondents ages 5-49 years, yielding a total sample of 14,251,279 individuals. In supplementary analyses, we limited the sample to respondents born 5, 4, 3, or 2 months before and after the school-entry age cutoff (December 31st) to maximize the comparability of early and late starters.

The 1989 Census covered all residents in Vietnam, including those usually resident in Vietnam, but who were overseas at the time of the Census; special groups, including the police force, army, and foreign affairs (de jure). Census day was April 1, 1989. The 1999 Census was conducted within 7 to 10 days of Census Day (April 1, 1999), and covered residents in Vietnam, including those usually resident in Vietnam, but who were overseas at the time of the census; special groups, including the police force, army and foreign affairs (de jure). The 2009 Census was conducted within 7 to 14 days of Census Day (April 1, 2009), and similarly covered residents in Vietnam, including those usually resident in Vietnam, but who were overseas at the time of the census; special groups, including the police force, army and foreign affairs (de jure).

The surveys provide information on demographic outcomes among all respondents, educational outcomes among respondents aged 5 years and older, and childbearing outcomes among women aged 15-49. The 1989, 1999, and 2009 Census contain data on month of birth based on the survey questions “Month and year of birth” [solar calendar month] (Census 1989), “In what solar calendar month and year was (Name) born?” (Census 1999), “In what solar calendar month and year was [the respondent] born?” (Census 2009) and was asked from all respondents. IPUMS harmonizes variables across surveys so that the codes have the same meaning across all surveys. Data on the month of birth was not available in the Vietnam Population and Housing Census of 2019, provided by IPUMS, and therefore not used in the current analysis.

Text S3. Education context in Vietnam

Education system

The Education Law of 1995 (Viet Nam National Assembly) describes the basic structure of the education system in Vietnam. The education system consists of five levels: preschool, primary school, secondary school, high school, and higher education. Basic education consists of five years of primary education, four years of secondary education, and three years of high school education. The government of Vietnam exercises tight control over development strategies. An increase in private sector engagement and transition to a market economy has brought in the need and focus to modernize the education system. Vietnam's government has devoted 15-20% of its spending budget to education since the late 1990s (Dang & Glewwe 2018). In 1990, Vietnam endorsed the Jomtien World Declaration on Education For All, which set the foundation for subsequent educational policies and reforms in Vietnam. Over 150 countries assembled in Jomtien, Thailand, and pledged to provide education for all by the year 2000. In 1991, the Vietnamese government introduced a law on compulsory schooling, the Law on Universal Primary Education. Vietnam has also participated in the EFA Fast Track Initiative (Global Partnership of Education) launched by the World Bank in 2002 to help low-income countries accelerate progress towards the EFA goal of enrolling all primary school-aged children in school by 2015. Enrollment in primary and secondary levels has been increasing since the early 1990s. While Vietnam's education system has made significant progress, challenges remain. While there is no gender gap at the primary school level, a gender gap appears at the upper-secondary school level and in urban vs. rural education.

School enrollment

The Education Law of 1998 requires that a child enroll in Grade 1 of primary school at the start of the academic year in the calendar year they become 6 years old (Viet Nam National Assembly). Since eligibility is based on calendar year, month of birth *within* a year of birth does not matter for grade 1 eligibility as opposed to month of birth *across* years of birth. A child born December in year of birth X, for example, is eligible whereas a child born in January in year of birth X+1 may not be eligible. Specifically, article 22 of Section 1 of the Education Law of 1998 stipulates that: "*Primary education is the compulsory level of education for all children from six to fourteen years old; it is conducted in five school years from the first to the fifth form. The age of pupils admitted to the first form is six years*". (Education Law of 1998) Similarly, the Education Law of 2019 also stipulates that "the entry age for the first grade is 6".

The school year starts in the first week of September and runs until the end of May the following calendar year. Parents are required to register their children for Grade 1 at local schools, and the registration process typically includes providing documents such as birth certificates and proof of residency. As a result of the school-entry age policy, children who are born just before December 31st start school one year earlier compared to children who are born just after December 31st. The policy has been suggested to be in place since 1945 when the nation became independent, although implementation and enforcement of the policy may have evolved over time. Lunar New Year in Vietnam does not coincide with December 31st on the Gregorian calendar because it follows the lunar calendar, meaning the new year falls on the first day of the lunar month (which usually lands sometime between late January and early February depending on the year).

Figure S1. Heterogeneity: impacts on education by gender

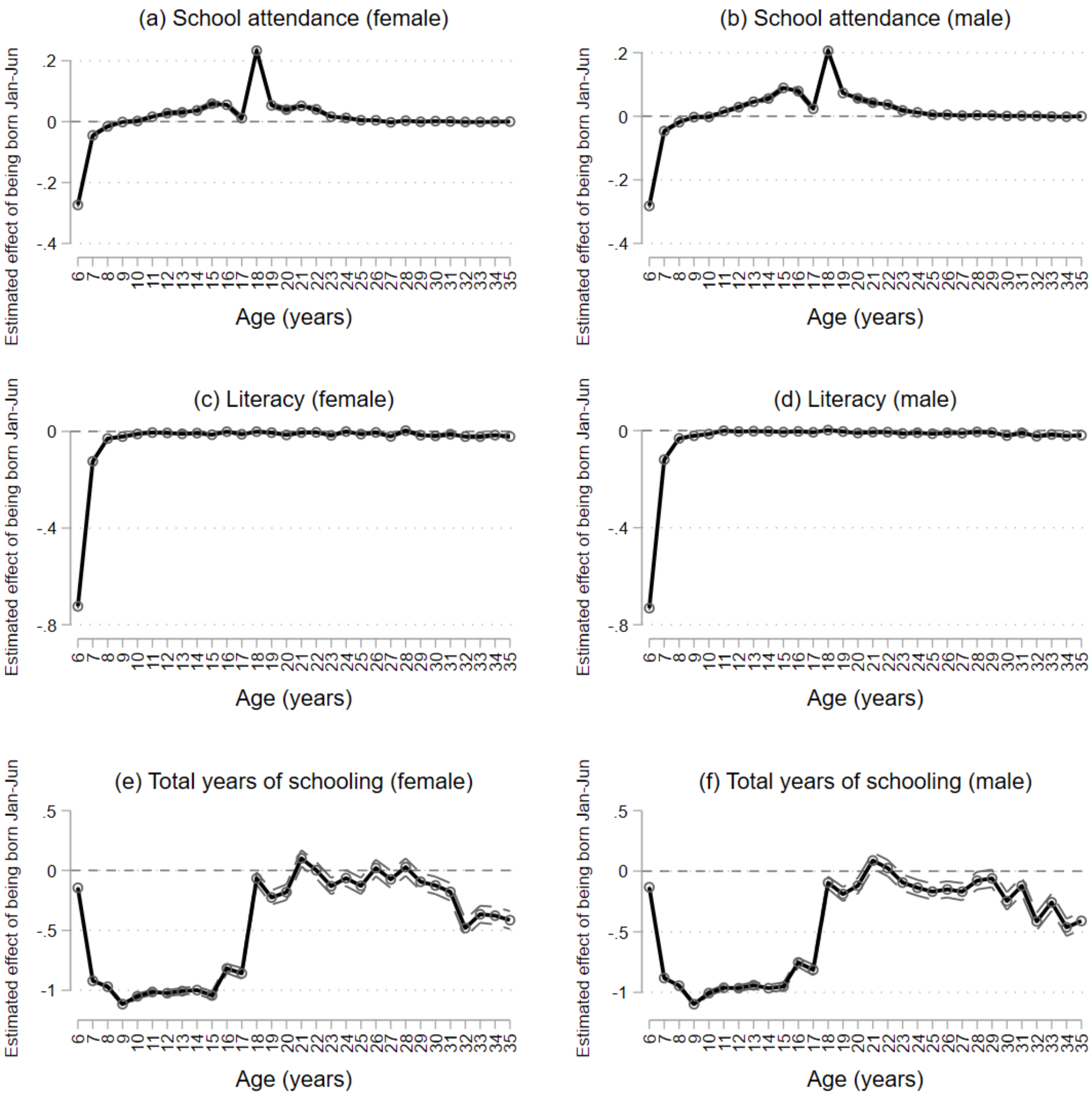

Notes: Figure shows intention-to-treat regression estimates for the effect of being born after the December 31st school-entry age cutoff on education by gender, estimated separately at each age. All models included the treatment variable (an indicator for being born between January and June), centered month of birth, and indicators for the Census year. The outcomes were a binary indicator for current school attendance (Figures (a) and (b)), literacy (Figures (c) and (d)), and total years of schooling completed (Figures (e) and (f)). Literacy in the Census data indicates whether the respondent could read and write in any language. A person was typically considered literate if he or she could both read and write. In addition, in 1999, people with 5 or more years of schooling were considered literate. In 2009, people who completed more than primary education were considered literate. Dashed lines represent 95% confidence intervals. The sample includes all respondents aged 6-35 years in the Vietnam Population and Housing Census of 1989, 1999, and 2009. (*N*=10,485,714).

Figure S2. Distribution in month of birth

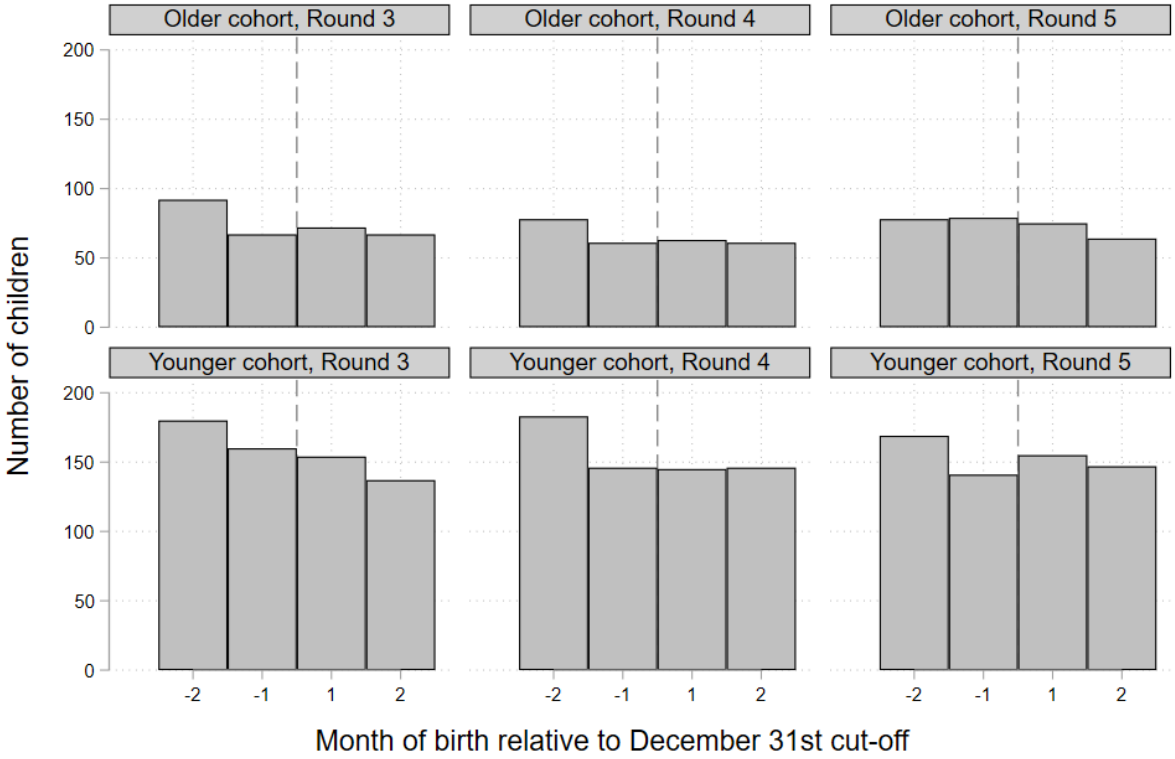

Notes: Figure shows the distribution in month of birth, separately by Young Lives cohort and survey round. The older cohort was born 1994-1995 and younger cohort was born 2001-2002. Sample includes respondents born two months before and after the December 31st cutoff in the Vietnam Young Lives study rounds 3-5.

Figure S3. Balance in background characteristics

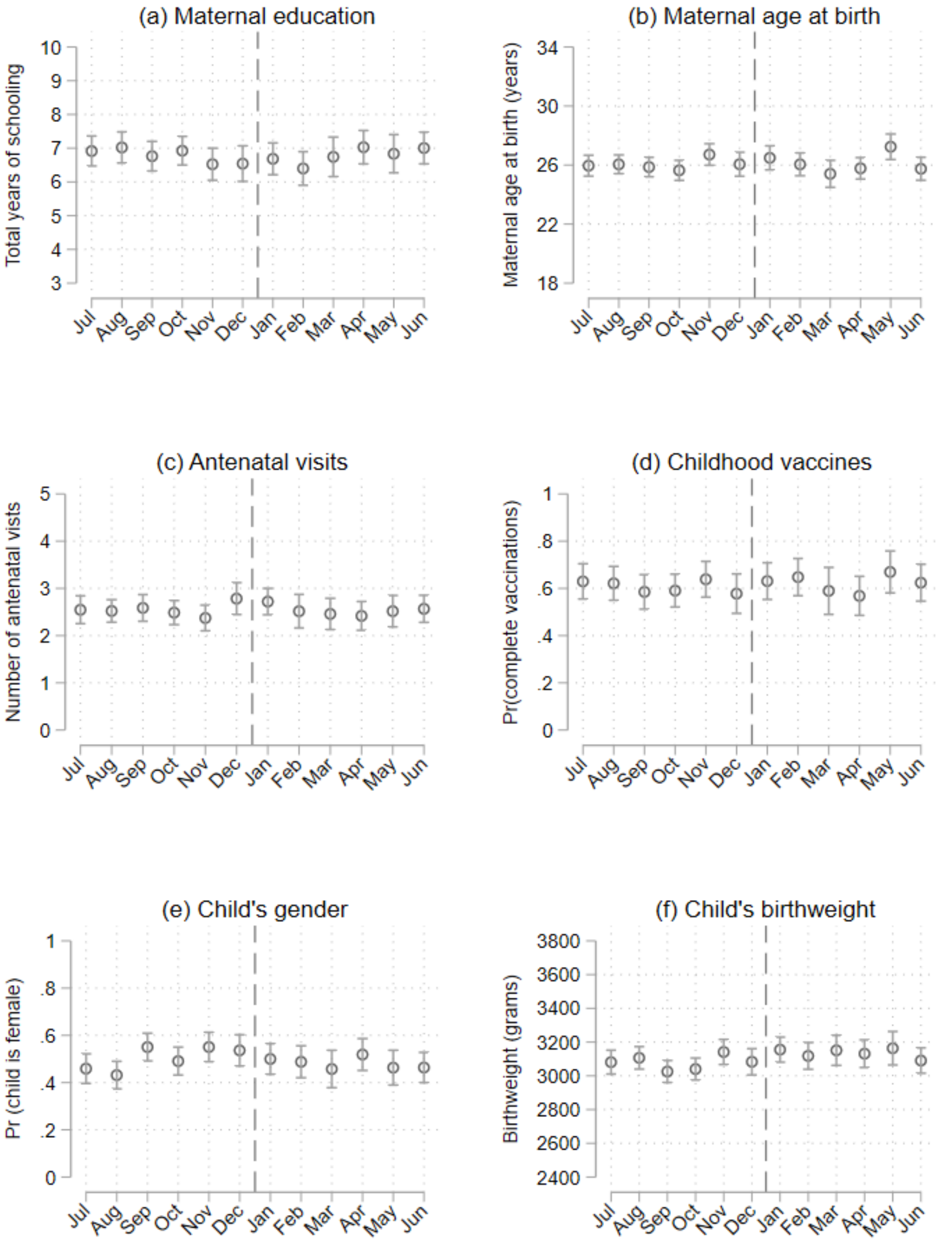

Notes: Figure shows means and confidence intervals for selected background characteristics by month of birth among respondents in the Young Lives Study. Outcomes were defined as maternal educational attainment (total years of schooling completed); maternal age at birth (years); number of maternal antenatal visits; a binary indicator for having completed all childhood vaccinations; an indicator for children's gender (female); and children's birthweight (grams). Sample includes respondents ages 14-23 years in the Vietnam Young Lives Study round 5 (*N*=2,682).

Figure S4. Placebo test: measured height by month of birth

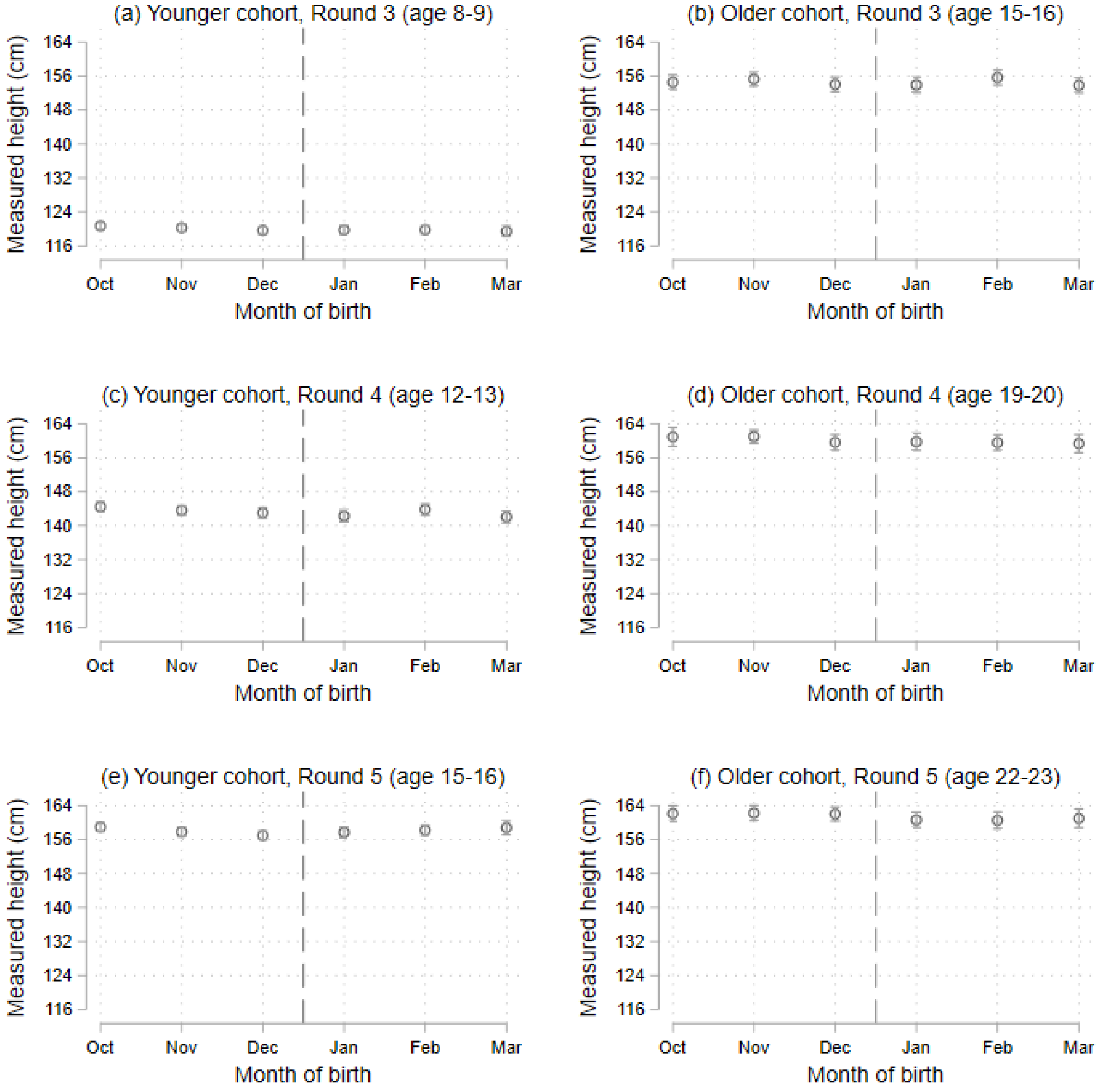

Notes: Figure shows measured height in centimeters by month of birth. Height was measured twice standing up on a height board. If there was a large difference between two measurements, children were measured one more time and the most common measurement was recorded. Sample includes respondents in the Young Lives Study. Figure (a) includes respondents in the younger cohort during round 3 who were ages 8-9 years old (N=924). Figure (b) includes respondents in the older cohort during round 3 who were ages 15-16 years old (N=440). Figure (c) includes respondents in the younger cohort during round 4 who were ages 12-13 years old (N=930). Figure (d) includes respondents in the older cohort during round 4 who were ages 19-20 years old (N=380). Figure (e) includes respondents in the younger cohort during round 5 who were ages 15-16 years old (N=909). Figure (f) includes respondents in the older cohort during round 5 who were ages 22-23 years old (N=427).

Figure S5. School enrollment by exact date of birth

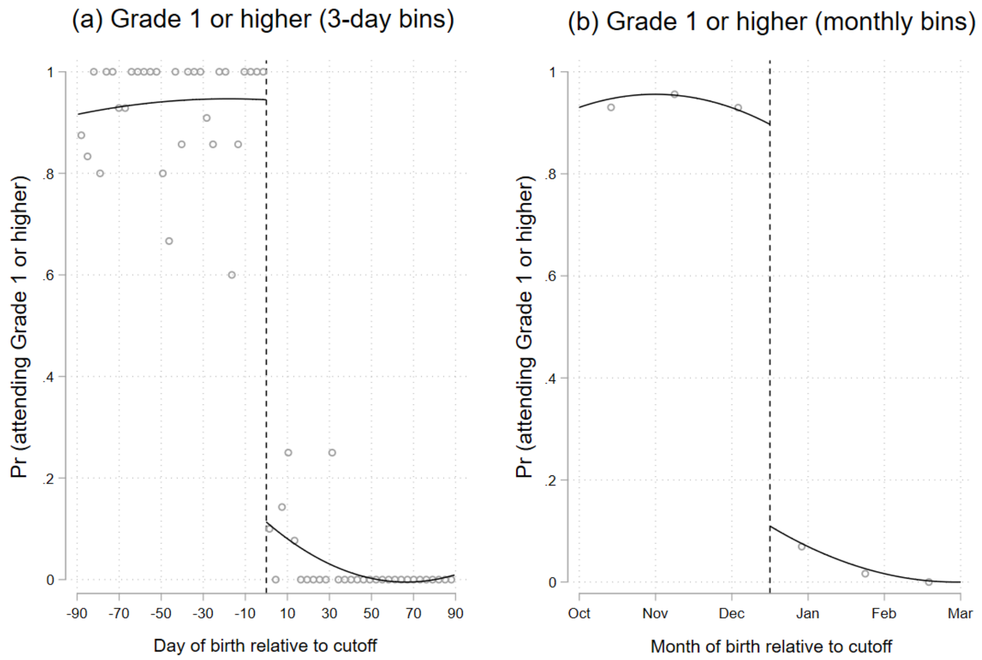

Notes: Figure shows the probability of attending Grade 1 or higher using 3-day bins (a) and monthly bins (b). The sample includes all children ages 5 at the beginning of the school year born to eligible mothers using the full birth history from the Vietnam Multiple Indicator Cluster Survey of 2020-21 (90 days +/- relative to the December 31st cut-off). The Multiple Indicator Cluster Survey is a nationally representative household survey developed by UNICEF that collects data on the status and well-being of women and children. Because eligible women in the Multiple Indicator Cluster Survey are ages 15-49 years, data on exact dates of birth were available for the next generation ages 0-35 years. In Vietnam, the school-entry age cut-off is defined as December 31st shown as a vertical dashed line. (*N*=418)

Table S1. First stage regression results: impacts on school-entry age (OLS)

First stage model	**(1)**	**(2)**	**(3)**	**(4)**	**(5)**
Dependent variable (DV): ***Age at start of grade 1 (years)***	**Without control variables**	**Adding age and gender**	**Adding age, gender, month of birth**	**Adding age indicators, gender, month of birth**	**Adding year of birth, gender, month of birth**
Predictor					
Born January-March (1=yes, 0=no)	0.698***	0.682***	0.473***	0.464***	0.794***
	(0.035)	(0.034)	(0.069)	(0.068)	(0.085)
Additional covariates					
Age in years (continuous)	-	✓	✓	-	-
Indicators for single-year age group	-	-	-	✓	-
Indicator for gender	-	✓	✓	✓	✓
Month of birth (continuous)	-	-	✓	✓	✓
Year of birth (continuous)	-	-	-	-	✓
Indicator for Young Lives cohort	-	-	-	-	✓
Data source and sample					
Young Lives Study (Round 5)	✓	✓	✓	✓	✓
Born between October-March	✓	✓	✓	✓	✓
Mean DV, October-December birth cohorts	5.1	5.1	5.1	5.1	5.1
Observations	1,334	1,334	1,334	1,334	1,334
R-squared	0.224	0.274	0.280	0.292	0.291

Notes: Table shows ordinary least regression results for the "first stage" relationship between being born after December 31st and age at school entry (years). The treatment variable was an indicator for whether the respondent was born between January and March and zero otherwise. Model 1 includes no covariates, Model 2 includes a continuous term in age and gender; Model 3 includes age, gender, and month of birth (continuous); Model 4 includes single-year indicators for age, gender, and month of birth; and Model 5 includes year of birth, month of birth, and gender. Robust unclustered standard errors in parentheses. The sample includes all respondents ages 15-23 years in the Young Lives Study Round 5 conducted in 2016 (N=1,334). *** p<0.01.

Table S2. Balance: estimates for background characteristics

Intention-to-treat model	**(1)**	**(2)**	**(3)**	**(4)**	**(5)**	**(6)**
Dependent variable (DV)	**Maternal education (years)**	**Maternal age at birth (years)**	**Number of antenatal visits**	**Childhood vaccines completed (1=yes)**	**Child's gender female (=1)**	**Child's birthweight (grams)**
Predictor						
Born January-June (1=yes)	0.027	-0.038	0.015	0.034	-0.050	51.4
	(0.292)	(0.470)	(0.187)	(0.048)	(0.039)	(45.9)
Additional covariates						
Month of birth	✓	✓	✓	✓	✓	✓
Age (years)	✓	✓	✓	✓	✓	✓
Gender	✓	✓	✓	✓	-	✓
Mean DV, Jul-Dec birth cohorts	6.8	26.1	2.5	0.605	0.499	3076.3
Observations	2,682	2,680	1,808	1,741	2,682	1,599
R-squared	0.000	0.001	0.002	0.004	0.001	0.013

Notes: Table shows ordinary least squares regression estimates for the effect of being born after the December 31st school-entry age cutoff on selected balance characteristics. The treatment variable was an indicator for whether the respondent was born between January and June and zero otherwise. The month of birth variable is continuous and centered as the month of birth - 6.5; age (in years) is a continuous variable, and gender is a dummy variable. Outcomes were defined as maternal educational attainment (total years of schooling completed); maternal age at birth (years); number of maternal antenatal visits; a binary indicator for having completed all childhood vaccinations; an indicator for children's gender (female); and children's birthweight (grams). Robust unclustered standard errors in parentheses. Data on antenatal visits, childhood vaccines, and birthweight were not available for the older Young Lives cohort. Sample includes respondents ages 14-23 years in the Vietnam Young Lives Study round 5 (*N*=2,682).

Table S3. Robustness checks: using narrower windows of month of birth

Intention-to-treat model	**(1)**	**(2)**	**(3)**	**(4)**	**(5)**
Dependent variable (DV): Number of children ever born	**+/- 6 months of birth around cutoff**	**+/- 5 months of birth around cutoff**	**+/- 4 months of birth around cutoff**	**+/- 3 months of birth around cutoff**	**+/- 2 months of birth around cutoff**
Predictor					
Born January-June (1=yes)	-0.095***	-0.104***	-0.115***	-0.065***	-0.112***
	(0.003)	(0.003)	(0.004)	(0.005)	(0.006)
Additional covariates					
Month of birth	✓	✓	✓	✓	✓
Age (years)	✓	✓	✓	✓	✓
Census year	✓	✓	✓	✓	✓
Sample					
Ages 24-49	✓	✓	✓	✓	✓
Female	✓	✓	✓	✓	✓
Mean DV, Jul-Dec birth cohorts	2.2	2.2	2.2	2.2	2.1
Observations	3,650,035	2,952,921	2,292,273	1,698,350	977,995
R-squared	0.230	0.232	0.234	0.237	0.239

Notes: Table shows ordinary least squares regression estimates for the effect of being born after the December 31st school-entry age cutoff on childbearing in the Census data. The treatment variable was an indicator for whether the respondent was born between January and June and zero otherwise. All models include the running variable month of birth (continuously), age (continuously in years) and indicators for census year. The running variable month of birth is centered as month of birth – 6.5. Robust unclustered standard errors in parentheses. Model 1 includes the sample born +/- 6 months around the December 31st school-entry age cutoff. Model 2 includes the sample born +/- 5 months around the December 31st school-entry age cutoff. Model 3 includes the sample born +/- 4 months around the December 31st school-entry age cutoff. Model 4 includes the sample born +/- 3 months around the December 31st school-entry age cutoff. Model 5 includes the sample born +/- 2 months around the December 31st school-entry age cutoff. Sample includes respondents ages 24-49 years at the time of the survey in the Vietnam Population and Housing Census 1989, 1999, and 2009 with complete data on childbearing outcomes (N=3,650,035). *** $p<0.01$

Table S4. Robustness checks: using additional control variables

Intention-to-treat model	**(1)**	**(2)**	**(3)**	**(4)**	**(5)**
Dependent variable (DV)	**Ever given birth (1=yes)**	**Two children or more (1=yes)**	**Three children or more (1=yes)**	**Number of children ever born**	**Ever married or cohabited (1=yes)**
Predictor					
Born January-June (1=yes)	-0.028***	-0.035***	-0.013***	-0.075***	-0.019***
	(0.001)	(0.001)	(0.001)	(0.003)	0.000
Additional covariates					
Month of birth	✓	✓	✓	✓	✓
Indicators for age (years)	✓	✓	✓	✓	✓
Indicators for ethnicity	✓	✓	✓	✓	✓
Census year	✓	✓	✓	✓	✓
Gender	-	-	-	-	✓
Sample					
Ages 24-49	✓	✓	✓	✓	✓
Female	✓	✓	✓	✓	✓
Male	-	-	-	-	✓
Mean DV, Jul-Dec birth cohorts	0.875	0.686	0.324	2.2	0.884
Observations	3,650,035	3,650,035	3,650,035	3,650,035	7,175,064
R-squared	0.092	0.197	0.170	0.229	0.128

Notes: Table shows ordinary least squares regression estimates for the effect of being born after the December 31st school-entry age cutoff on long-run family formation in the Census data when using additional control variables. The treatment variable was an indicator for whether the respondent was born between January and June and zero otherwise. All models include the running variable month of birth (continuously), indicators for age, indicators for ethnicity, and indicators for census year. Model 5 additionally controlled for gender. The running variable month of birth is centered as month of birth – 6.5. Robust unclustered standard errors in parentheses. Sample includes respondents ages 24-49 years at the time of the survey in the Vietnam Population and Housing Census 1989, 1999, and 2009 with complete data on childbearing (*N*=7,175,064). *** p<0.01

Table S5. Robustness checks: respondents born in or after 1970

Intention-to-treat model	**(1)**	**(2)**	**(3)**	**(4)**	**(5)**
Dependent variable (DV)	**Ever given birth (1=yes)**	**Two children or more (1=yes)**	**Three children or more (1=yes)**	**Number of children ever born**	**Ever married or cohabited (1=yes)**
Predictor					
Born January-June (1=yes)	-0.049***	-0.065***	-0.017***	-0.133***	-0.037***
	(0.001)	(0.001)	(0.001)	(0.003)	(0.001)
Additional covariates					
Month of birth	✓	✓	✓	✓	✓
Age (years)	✓	✓	✓	✓	✓
Census year	✓	✓	✓	✓	✓
Gender	-	-	-	-	✓
Sample					
Ages 24-39	✓	✓	✓	✓	✓
Female	✓	✓	✓	✓	✓
Male	-	-	-	-	✓
Mean DV, Jul-Dec birth cohorts	0.837	0.576	0.169	1.7	0.835
Observations	1,871,665	1,871,665	1,871,665	1,871,665	3,720,644
R-squared	0.073	0.169	0.079	0.171	0.107

Notes: Table shows ordinary least squares regression estimates for the effect of being born after the December 31st school-entry age cutoff on long-run family formation in the Census data. The treatment variable was an indicator for whether the respondent was born between January and June and zero otherwise. All models include the running variable month of birth (continuously), age (continuously in years) and indicators for census year. Model 5 additionally controlled for gender. The running variable month of birth is centered as month of birth – 6.5. Robust unclustered standard errors in parentheses. Sample includes respondents born in or after 1970 (ages 24-39 years) in the Vietnam Population and Housing Census 1989, 1999, and 2009 with complete data on childbearing (*N*=7,175,064). *** p<0.01

Table S6. Robustness checks: analyzing 2009 Census separately

Intention-to-treat model	**(1)**	**(2)**	**(3)**	**(4)**	**(5)**
Dependent variable (DV)	**Ever given birth (1=yes)**	**Two children or more (1=yes)**	**Three children or more (1=yes)**	**Number of children ever born**	**Ever married or cohabited (1=yes)**
Predictor					
Born January-June (1=yes)	-0.032***	-0.044***	-0.016***	-0.096***	-0.022***
	(0.001)	(0.001)	(0.001)	(0.003)	(0.001)
Additional covariates					
Month of birth	✓	✓	✓	✓	✓
Age (years)	✓	✓	✓	✓	✓
Gender	-	-	-	-	✓
Sample					
Ages 24-49	✓	✓	✓	✓	✓
Female	✓	✓	✓	✓	✓
Male	-	-	-	-	✓
Mean DV, Jul-Dec birth cohorts	0.879	0.682	0.288	2.1	0.886
Observations	2,792,143	2,792,143	2,792,143	2,792,143	5,521,351
R-squared	0.055	0.148	0.149	0.195	0.089

Notes: Table shows ordinary least squares regression estimates for the effect of being born after the December 31st school-entry age cutoff on long-run family formation in the Census data. The treatment variable was an indicator for whether the respondent was born between January and June and zero otherwise. All models include the running variable month of birth (continuously), age (continuously in years). Model 5 additionally controlled for gender. The running variable month of birth is centered as month of birth – 6.5. Robust unclustered standard errors in parentheses. Sample includes respondents ages 24-49 years at the time of the survey in the Vietnam Population and Housing Census 2009 with complete data on childbearing (*N*=5,521,351). *** p<0.01

Table S7. Robustness checks: analyzing 1999 Census separately

Intention-to-treat model	**(1)**	**(2)**	**(3)**	**(4)**	**(5)**
Dependent variable (DV)	**Ever given birth (1=yes)**	**Two children or more (1=yes)**	**Three children or more (1=yes)**	**Number of children ever born**	**Ever married or cohabited (1=yes)**
Predictor					
Born January-June (1=yes)	-0.029***	-0.036***	-0.017***	-0.085***	-0.021***
	(0.002)	(0.003)	(0.003)	(0.010)	(0.001)
Additional covariates					
Month of birth	✓	✓	✓	✓	✓
Age (years)	✓	✓	✓	✓	✓
Gender	-	-	-	-	✓
Sample					
Ages 24-49	✓	✓	✓	✓	✓
Female	✓	✓	✓	✓	✓
Male	-	-	-	-	✓
Mean DV, Jul-Dec birth cohorts	0.870	0.692	0.389	2.4	0.880
Observations	440,884	440,884	440,884	440,884	855,162
R-squared	0.044	0.145	0.197	0.234	0.078

Notes: Table shows ordinary least squares regression estimates for the effect of being born after the December 31st school-entry age cutoff on long-run family formation in the Census data. The treatment variable was an indicator for whether the respondent was born between January and June and zero otherwise. All models include the running variable month of birth (continuously), age (continuously in years). Model 5 additionally controlled for gender. The running variable month of birth is centered as month of birth – 6.5. Robust unclustered standard errors in parentheses. Sample includes respondents ages 24-49 years at the time of the survey in the Vietnam Population and Housing Census 1999 with complete data on childbearing (*N*=855,162). *** p<0.01

Table S8. Robustness checks: analyzing 1989 Census separately

Intention-to-treat model	**(1)**	**(2)**	**(3)**	**(4)**	**(5)**
Dependent variable (DV)	**Ever given birth (1=yes)**	**Two children or more (1=yes)**	**Three children or more (1=yes)**	**Number of children ever born**	**Ever married or cohabited (1=yes)**
Predictor					
Born January-June (1=yes)	-0.028***	-0.036***	-0.028***	-0.136***	-0.020***
	(0.002)	(0.003)	(0.003)	(0.013)	(0.002)
Additional covariates					
Month of birth	✓	✓	✓	✓	✓
Age (years)	✓	✓	✓	✓	✓
Gender	-	-	-	-	✓
Sample					
Ages 24-49	✓	✓	✓	✓	✓
Female	✓	✓	✓	✓	✓
Male	-	-	-	-	✓
Mean DV, Jul-Dec birth cohorts	0.853	0.708	0.502	2.9	0.871
Observations	417,008	417,008	417,008	417,008	798,551
R-squared	0.049	0.120	0.203	0.287	0.062

Notes: Table shows ordinary least squares regression estimates for the effect of being born after the December 31st school-entry age cutoff on long-run family formation in the Census data. The treatment variable was an indicator for whether the respondent was born between January and June and zero otherwise. All models include the running variable month of birth (continuously), age (continuously in years). Model 5 additionally controlled for gender. The running variable month of birth is centered as month of birth – 6.5. Robust unclustered standard errors in parentheses. Sample includes respondents ages 24-49 years at the time of the survey in the Vietnam Population and Housing Census 1989 with complete data on childbearing (*N*= 855,162). *** p<0.01

Table S9. Robustness checks: adding (Born Jan-Jun) x Birth month (Young Lives)

Intention-to-treat model	**(1)**	**(2)**	**(3)**	**(4)**	**(5)**
Dependent variable (DV): Currently attending school (=1)	**Base specification**	**Adding (Born Jan-Jun)xMOB**	**Adding (Born Jan-Jun)xMOBx Gender**	**Adding AgexGender**	**Adding indicator for YL Birth Cohort**
Predictor					
Born January-June (1=yes)	0.033**	0.042**	0.043**	0.042**	0.038**
	(0.013)	(0.017)	(0.018)	(0.018)	(0.017)
Additional covariates					
Month of birth (MOB)	✓	✓	✓	✓	✓
Age (years)	✓	✓	✓	✓	✓
Gender	✓	✓	✓	✓	✓
(Born January-June)xMOB	-	✓	✓	✓	✓
(Born January-June)xMOBxGender	-	-	✓	✓	✓
AgexGender	-	-	-	✓	✓
YL Birth Cohort, Young (=1)	-	-	-	-	✓
Mean DV, Jul-Dec birth cohorts	0.811	0.811	0.811	0.811	0.811
Observations	10,746	10,746	10,746	10,746	10,746
Respondents	2,838	2,838	2,838	2,838	2,838

Notes: Table shows ordinary least squares regression estimates for the effect of being born after the December 31st school-entry age cutoff on school attendance. The treatment variable was an indicator for whether the respondent was born between January and June and zero otherwise. The month of birth (MOB) variable is continuous and centered as the month of birth - 6.5; age (in years) is a continuous variable, and Census year and gender are dummy variables. (Born January-June) x month of birth (MOB) is the interaction between an indicator for being born between January and June and a linear function of centered month of birth. Column 5 adds an indicator for being in either the young cohort or old cohort of the Young Lives (YL) Study. Robust unclustered standard errors in parentheses. Data from the Young Lives Study (Rounds 2-5), 2006-2017. *** p<0.01, ** p<0.05, *p<0.10.

Table S10. Robustness checks: adding (Born Jan-Jun) x Birth month (Census, ages 15-23)

Intention-to-treat model	**(1)**	**(2)**	**(3)**	**(4)**	**(5)**
Dependent variable (DV)	**Currently attending school (1=yes)**	**Ever given birth (1=yes)**	**Number of children ever born**	**Number of own children in household**	**Ever married or cohabited (1=yes)**
Predictor					
Born January-June (1=yes)	0.062***	-0.048***	-0.065***	-0.044***	-0.043***
	(0.001)	(0.001)	(0.002)	(0.001)	(0.001)
Additional covariates					
Month of birth (MOB)	✓	✓	✓	✓	✓
(Born January-June)xMOB	✓	✓	✓	✓	✓
Age (years)	✓	✓	✓	✓	✓
Census year	✓	✓	✓	✓	✓
Gender	✓	-	-	✓	✓
Sample					
Ages 15-23	✓	✓	✓	✓	✓
Female	✓	✓	✓	✓	✓
Male	✓	-	-	✓	✓
Mean DV, Jul-Dec birth cohorts	0.323	0.188	0.243	0.157	0.196
Observations	3,367,641	1,656,126	1,656,126	3,367,641	3,367,641
R-squared	0.237	0.205	0.184	0.145	0.214

Notes: Table shows ordinary least squares regression estimates for the effect of being born after the December 31st school-entry age cutoff on school attendance and early family formation. The treatment variable was an indicator for whether the respondent was born between January and June and zero otherwise. The month of birth (MOB) variable is continuous and centered as the month of birth - 6.5; age (in years) is a continuous variable, and Census year and gender are dummy variables. (Born Jan-Jun) x Birth month is the interaction between an indicator for being born between January and June and a linear function of centered month of birth. Robust unclustered standard errors in parentheses. Sample of respondents aged 15-23 at the time of the survey in the Vietnam Population and Housing Census 1989, 1999, and 2009 with complete information on childbearing outcomes (N=3,367,641). *** p<0.01, ** p<0.05, *p<0.10.

Table S11. Robustness checks: adding (Born Jan-Jun) x Birth month (Census, ages 24-49)

Intention-to-treat model	**(1)**	**(2)**	**(3)**	**(4)**	**(5)**
Dependent variable (DV)	**Ever given birth (1=yes)**	**Two children or more (1=yes)**	**Three children or more (1=yes)**	**Number of children ever born**	**Ever married or cohabited (1=yes)**
Predictor					
Born January-June (1=yes)	-0.030***	-0.039***	-0.014***	-0.083***	-0.020***
	(0.001)	(0.001)	(0.001)	(0.003)	(0.001)
Additional covariates					
Month of birth (MOB)	✓	✓	✓	✓	✓
(Born January-June)xMOB	✓	✓	✓	✓	✓
Age (years)	✓	✓	✓	✓	✓
Census year	✓	✓	✓	✓	✓
Gender	✓	-	-	✓	✓
Sample					
Ages 24-49	✓	✓	✓	✓	✓
Female	✓	✓	✓	✓	✓
Male	-	-	-	-	✓
Mean DV, Jul-Dec birth cohorts	0.875	0.686	0.324	2.2	0.884
Observations	3,648,177	3,648,177	3,648,177	3,648,177	7,172,949
R-squared	0.054	0.146	0.181	0.231	0.084

Notes: Table shows ordinary least squares regression estimates for the effect of being born after the December 31st school-entry age cutoff on childbearing and marriage. The treatment variable was an indicator for whether the respondent was born between January and June and zero otherwise. The month of birth (MOB) variable is continuous and centered as the month of birth - 6.5; age (in years) is a continuous variable, and Census year and gender are dummy variables. (Born Jan-Jun) x Birth month is the interaction between an indicator for being born between January and June and a linear function of centered month of birth. Robust unclustered standard errors in parentheses. Sample of respondents aged 24-49 at the time of the survey in the Vietnam Population and Housing Census 1989, 1999, and 2009 with complete information on childbearing outcomes (N=7,175,064). *** p<0.01, ** p<0.05, *p<0.10.

Table S12. Impact on lifetime prevalence of offspring mortality

Intention-to-treat model	**(1)**	**(2)**
Dependent variable (DV): ***Any offspring mortality (=1)***	**Subsample ages 18-23 years**	**Subsample ages 24-49 years**
Predictor		
Born January-June (1=yes)	-0.002***	-0.004***
	(0.0003)	(0.0005)
Additional covariates		
Month of birth	✓	✓
Age (years)	✓	✓
Census year	✓	✓
Sample		
Female	✓	✓
Mean DV, Jul-Dec birth cohorts	0.008	0.051
Observations	1,058,588	3,649,623
R-squared	0.005	0.033

Notes: Table shows ordinary least squares regression estimates for the effect of being born after the December 31st school-entry age cutoff on lifetime prevalence of experiencing any offspring mortality among women in the Census data. The treatment variable was an indicator for whether the respondent was born between January and June and zero otherwise. All models include the running variable month of birth (continuously), age (continuously in years) and indicators for census year. The running variable month of birth is centered as month of birth – 6.5. Robust unclustered standard errors in parentheses. Sample includes all women ages 18-23 years (model 1) or 24-49 years (model 2) at the time of the survey in the Vietnam Population and Housing Census 1989, 1999, and 2009 with complete data on childbearing outcomes (*N*= 4,708,211). *** p<0.01

Table S13. Alternative empirical approach: within-household analyses

Intention-to-treat model	**(1)**	**(2)**	**(3)**	**(4)**	**(5)**
Dependent variable (DV)	**Ever given birth (1=yes)**	**Two children or more (1=yes)**	**Three children or more (1=yes)**	**Number of children ever born**	**Ever married or cohabited (1=yes)**
Predictor					
Born January-June (1=yes)	-0.046***	-0.057***	-0.037***	-0.192***	-0.023***
	(0.004)	(0.003)	(0.003)	(0.012)	(0.001)
Additional covariates					
Month of birth	✓	✓	✓	✓	✓
Age (years)	✓	✓	✓	✓	✓
Census year	✓	✓	✓	✓	✓
Gender	-	-	-	-	✓
Household fixed effects	✓	✓	✓	✓	✓
Sample					
Ages 24-49	✓	✓	✓	✓	✓
Female	✓	✓	✓	✓	✓
Male	-	-	-	-	✓
Number of households	3,408,765	3,408,765	3,408,765	3,408,765	3,927,285
N, all households	3,666,413	3,666,413	3,666,413	3,666,413	7,176,722
N, households with >1 respondent	3,026,716	3,026,716	3,026,716	3,026,716	6,084,064
R-squared	0.163	0.360	0.428	0.425	0.165

Notes: Table shows ordinary least squares regression estimates for the effect of being born after the December 31st school-entry age cutoff on long-run family formation in the Census data when adding household fixed effects. The treatment variable was an indicator for whether the respondent was born between January and June and zero otherwise. All models include the running variable month of birth (continuously), age (continuously in years), indicators for census year, and household fixed effects. Model 5 additionally controls for gender. The running variable month of birth is centered as month of birth – 6.5. Robust unclustered standard errors in parentheses. Sample includes respondents ages 24-49 years at the time of the survey in the Vietnam Population and Housing Census 1989, 1999, and 2009 with complete data on childbearing outcomes (N=7,176,722). *** $p<0.01$

Table S14. Pathways: controlling for school attendance

Intention-to-treat model	(1)	(2)	(3)	(4)	(5)
Dependent variable (DV)	**Currently attending school (1=yes)**	**Ever given birth (1=yes)**	**Number of children ever born**	**Number of own children in household**	**Ever married or cohabited (1=yes)**
	Panel A. RDD estimates without controlling for school attendance				
Predictor					
Born January-June (1=yes)	0.150***	-0.020***	-0.020***	-0.013***	-0.025***
	(0.003)	(0.002)	(0.003)	(0.001)	(0.002)
Additional covariates					
Month of birth	✓	✓	✓	✓	✓
Age (years)	✓	✓	✓	✓	✓
Census year	✓	✓	✓	✓	✓
Gender	✓	-	-	✓	✓
Sample					
Ages 17-18	✓	✓	✓	✓	✓
Female	✓	✓	✓	✓	✓
Male	✓	-	-	✓	✓
	Panel B. RDD estimates controlling for current school attendance				
Predictor					
Born January-June (1=yes)	-	-0.006***	-0.006**	-0.006***	-0.008***
	-	(0.002)	(0.002)	(0.001)	(0.002)
Additional covariates					
Month of birth	-	✓	✓	✓	✓
Age (years)	-	✓	✓	✓	✓
Census year	-	✓	✓	✓	✓
Gender	-	-	-	✓	✓
School attendance (1=yes)	-	✓	✓	✓	✓
Sample					
Ages 17-18	-	✓	✓	✓	✓
Female	-	✓	✓	✓	✓
Male	-	-	-	✓	✓
Mean DV, Jul-Dec birth cohorts	0.315	0.067	0.073	0.043	0.096
Observations	385,042	184,682	184,682	385,042	385,042

Notes: Table shows ordinary least squares regression estimates for the effect of being born after the December 31st school-entry age cutoff on early family formation in the Census data. The treatment variable was an indicator for whether the respondent was born between January and June and zero otherwise. All models include the running variable month of birth (continuously), age (continuously in years), and indicators for census year. Models 4 and 5 additionally control for gender. Panel B additional controls for current school attendance (binary). The running variable month of birth is centered as month of birth – 6.5. Robust unclustered standard errors in parentheses. Sample includes respondents ages 17-18 years at the time of the survey in the Vietnam Population and Housing Census 1989, 1999, and 2009 with complete data on childbearing outcomes (*N*=369,974). ** p<0.05, *** p<0.01

Table S15. Pathways: controlling for years of schooling

Intention-to-treat model	**(1)**	**(2)**	**(3)**	**(4)**	**(5)**
Dependent variable (DV)	**Currently attending school (1=yes)**	**Ever given birth (1=yes)**	**Number of children ever born**	**Number of own children in household**	**Ever married or cohabited (1=yes)**
	Panel A. RDD estimates without controlling for years of schooling				
Predictor					
Born January-June (1=yes)	0.150***	-0.020***	-0.020***	-0.013***	-0.025***
	(0.003)	(0.002)	(0.003)	(0.001)	(0.002)
Additional covariates					
Month of birth	✓	✓	✓	✓	✓
Age (years)	✓	✓	✓	✓	✓
Census year	✓	✓	✓	✓	✓
Gender	✓	-	-	✓	✓
Sample					
Ages 17-18	✓	✓	✓	✓	✓
Female	✓	✓	✓	✓	✓
Male	✓	-	-	✓	✓
	Panel B. RDD estimates controlling for years of schooling				
Predictor					
Born January-June (1=yes)	-	-0.022***	-0.023***	-0.015***	-0.029***
	-	(0.002)	(0.002)	(0.001)	(0.002)
Additional covariates					
Month of birth	-	✓	✓	✓	✓
Age (years)	-	✓	✓	✓	✓
Census year	-	✓	✓	✓	✓
Gender	-	-	-	✓	✓
Years of schooling	-	✓	✓	✓	✓
Sample					
Ages 17-18	-	✓	✓	✓	✓
Female	-	✓	✓	✓	✓
Male	-	-	-	✓	✓
Mean DV, Jul-Dec birth cohorts	0.315	0.067	0.073	0.043	0.096
Observations	385,042	184,682	184,682	385,042	385,042

Notes: Table shows ordinary least squares regression estimates for the effect of being born after the December 31st school-entry age cutoff on early family formation in the Census data. The treatment variable was an indicator for whether the respondent was born between January and June and zero otherwise. All models include the running variable month of birth (continuously), age (continuously in years), and indicators for census year. Models 4 and 5 additionally control for gender. Panel B additional controls for total years of schooling completed. The running variable month of birth is centered as month of birth – 6.5. Robust unclustered standard errors in parentheses. Sample includes respondents ages 17-18 years at the time of the survey in the Vietnam Population and Housing Census 1989, 1999, and 2009 with complete data on childbearing outcomes (*N*=369,974). ** p<0.05, *** p<0.01

References for Appendix

Cavallo M, Dhuey E, Fumarco L, Halewyck L & ter Meulen S. (2026). The Economics of Age at School Entry: Insights from Evidence and Methods. (EdWorkingPaper: 26-1383). Retrieved from Annenberg Institute at Brown University: doi: 10.26300/ysjw-xd37.

Dhuey, E., Koebel, K (2022). Is there an optimal school starting age?. IZA World of Labor 2022: 247. doi: 10.15185/izawol.247.v2.

Favara M, Crivello G, Penny M, et al (2021). Cohort Profile Update: The Young Lives study. *International Journal of Epidemiology* 50(6): 1784-5e.

Dang HH, Glewwe PW (2018). Well Begun, But Aiming Higher: A Review of Vietnam's Education Trends in the Past 20 Years and Emerging Challenges. *J Dev Stud*. 54(7):1171-1195.

Liao J, Schröder H, Chin EK, et al (2023). The effect of school-entry age on health is understudied in low- and middle-income countries: A scoping review and future directions for research. *SSM Popul Health*. May 2;22:101423.

Minnesota Population Center. Integrated Public Use Microdata Series, International: Version 7.3 [dataset]. In: University of Minnesota, editor. Minneapolis, MN: IPUMS; 2020.

UNESCO Dakar Framework for Action, Education for All - 2000.

The Education Law of 1998. Viet Nam National Assembly.

The Education Law of 2019. Viet Nam National Assembly.
